# Model-driven analysis of mutant fitness experiments improves genome-scale metabolic models of *Zymomonas mobilis* ZM4

**DOI:** 10.1371/journal.pcbi.1008137

**Published:** 2020-08-17

**Authors:** Wai Kit Ong, Dylan K. Courtney, Shu Pan, Ramon Bonela Andrade, Patricia J. Kiley, Brian F. Pfleger, Jennifer L. Reed

**Affiliations:** 1 Department of Chemical and Biological Engineering, University of Wisconsin – Madison, Madison, Wisconsin, United States of America; 2 DOE Great Lakes Bioenergy Research Center, Univ. of Wisconsin-Madison – Madison, Madison, Wisconsin, United States of America; 3 Department of Biomolecular Chemistry, University of Wisconsin – Madison, Madison, Wisconsin, United States of America; The Pennsylvania State University, UNITED STATES

## Abstract

Genome-scale metabolic models have been utilized extensively in the study and engineering of the organisms they describe. Here we present the analysis of a published dataset from pooled transposon mutant fitness experiments as an approach for improving the accuracy and gene-reaction associations of a metabolic model for *Zymomonas mobilis* ZM4, an industrially relevant ethanologenic organism with extremely high glycolytic flux and low biomass yield. Gene essentiality predictions made by the draft model were compared to data from individual pooled mutant experiments to identify areas of the model requiring deeper validation. Subsequent experiments showed that some of the discrepancies between the model and dataset were caused by polar effects, mis-mapped barcodes, or mutants carrying both wild-type and transposon disrupted gene copies—highlighting potential limitations inherent to data from individual mutants in these high-throughput datasets. Therefore, we analyzed correlations in fitness scores across all 492 experiments in the dataset in the context of functionally related metabolic reaction modules identified within the model via flux coupling analysis. These correlations were used to identify candidate genes for a reaction in histidine biosynthesis lacking an annotated gene and highlight metabolic modules with poorly correlated gene fitness scores. Additional genes for reactions involved in biotin, ubiquinone, and pyridoxine biosynthesis in *Z*. *mobilis* were identified and confirmed using mutant complementation experiments. These discovered genes, were incorporated into the final model, *i*ZM4_478, which contains 747 metabolic and transport reactions (of which 612 have gene-protein-reaction associations), 478 genes, and 616 unique metabolites, making it one of the most complete models of *Z*. *mobilis* ZM4 to date. The methods of analysis that we applied here with the *Z*. *mobilis* transposon mutant dataset, could easily be utilized to improve future genome-scale metabolic reconstructions for organisms where these, or similar, high-throughput datasets are available.

## Author summary

We have used several methods of analysis of a high-throughput transposon library dataset for the organism *Zymomonas mobilis* in order to improve a newly developed genome-scale metabolic model. Data from individual experiments within this dataset were compared to predictions made by our draft model to determine areas of inaccuracies in the model. Correlations within the dataset were investigated in the framework of metabolic modules in the model enabling us to identify, and subsequently experimentally confirm, genes for several reactions in the model that previously lacked an associated gene. Finally, we looked at which metabolic modules correlate poorly, highlighting where metabolic knowledge gaps in *Z*. *mobilis* may still reside. Our metabolic model, *i*ZM4_478, is one of the most complete for *Z*. *mobilis* ZM4 to date, and we expect it will be a valuable tool for investigating the unique, streamlined metabolism of this organism. The data analysis approaches that we used can be easily applied by other investigators in the development of metabolic models for other organisms with similar datasets, leading to more complete assignment of genes to reactions and more accurate metabolic models.

## Introduction

*Zymomonas mobilis* is an aerotolerant, Gram-negative, alpha-proteobacterium known for its ethanol production capabilities and exceptionally high glycolytic flux. Glucose uptake through facilitated diffusion coupled with high expression of the Entner-Doudoroff (ED) pathway results in uptake rates exceeding most common organisms, such as *Escherichia coli*, by several fold [1]. *Z*. *mobilis* converts up to 98% of glucose, fructose, or sucrose to ethanol [2,3]. This streamlined metabolism, low-biomass yield, robust growth without oxygen, and the ability to fix atmospheric nitrogen [4] without loss of product yield makes *Z*. *mobilis* an industrially relevant microbe as a biofuel and biochemical producer. Previous metabolic engineering efforts have focused on expanding its substrates (e.g., xylose [5] and arabinose [6]), products (e.g., sorbitol [7] and β-carotene [8]), and tolerance to lignotoxins [9], and have been recently reviewed [10].

The genome of *Z*. *mobilis* ZM4 was first sequenced and annotated by Seo et al. [11] in 2005, improved by Yang et al. [12] in 2011, and recently updated with a higher degree of emphasis on native plasmids [13]. These genome annotations have enabled a systematic approach to study this organism via metabolic modeling. To date, there have been two medium-scale stoichiometric models [14,15], one kinetic model of the ED pathway in *Z*. *mobilis* [16], and four genome-scale metabolic models [17–20]. In addition to these models, metabolic flux analysis studies using ^13^C and ^31^P or ^2^H tracers have been applied to investigate the thermodynamics and central metabolic flux distributions in *Z*. *mobilis* ZM4 [21,22]. The genome-scale metabolic models describe the complete known metabolic network and can be used to explore the capabilities of *Z*. *mobilis*. However, for these predictions to be useful, the model must accurately portray the organism’s metabolism. One method for testing and improving the validity of these models is through the integration of datasets from high-throughput “-omics” experiments [23,24], including pooled transposon mutant fitness profiling experiments [25,26].

Transposon mutagenesis is a valuable genetic tool for generating large mutant libraries. Inclusion of a short DNA barcode into the transposon provides an easy way to map individual mutants to gene disruption events via barcode sequencing, an approach especially useful in high-throughput pooled fitness profiling experiments. The abundance of individual mutants can be quantified at the start and end of a growth experiment using barcode sequencing techniques such as Bar-seq [27], which enables the determination of a mutant’s, and thereby a gene’s, relative fitness under different experimental conditions. Such datasets have been generated for many organisms with an increasing number of conditions tested, and have proven useful for identifying mutant phenotypes and suggesting gene functions [25,28–31].

Pooled transposon mutant fitness profiling datasets represent a wealth of pooled *in vivo* phenotype data for mutants throughout the genome; however, these datasets have not been fully leveraged in the development or investigation of genome-scale metabolic models. Gene-protein-reaction (GPR) associations, identifiers incorporated in the model that link a gene encoding a protein to the respective reaction the protein catalyzes, provide a direct method for mapping the gene fitness data to metabolic reactions and pathways. Modeling methods such as flux balance analysis (FBA) can be used to interpret the results of these experiments by investigating fitness scores (the average log_2_ of the change in the abundance of a barcoded transposon for a given gene in an individual experiment or media condition) in the context of the metabolic network. Discrepancies between the high-throughput data and model predictions can then be used to correct errors in the model.

While data from individual experiments has been used to refine models, model informed investigation of gene fitness correlations across multiple conditions has yet to be applied in the same way. Flux coupling analysis [32] of the genome-scale model can be used to identify metabolic modules in which we expect the gene’s fitness scores to be highly correlated within the dataset. As an example, disruption of individual reactions within a linear pathway should result in the same phenotype, and therefore similar fitness scores, in each individual experiment in the dataset. While poorly correlating modules highlight potential knowledge gaps, well correlating modules can be used to identify candidates for genes responsible for module reactions that lack a GPR in the model.

We have developed a new genome-scale metabolic network model for *Z*. *mobilis* ZM4 (summary statistics given in Table 1). The model was used in conjunction with a dataset from pooled mutant fitness experiments for 1,586 genes across 492 different experiments [26]. Corrections were made to the original draft model by first comparing the data from two experiments in the dataset to predictions made by the model for growth on minimal media. Through analysis of the correlation of fitness scores for individual genes in the entire pooled mutant dataset, and our model-enabled gene search (MEGS) approach [33], candidate genes were identified for reactions in histidine, biotin, ubiquinone, and pyridoxine biosynthesis pathways. These gene candidates were then experimentally verified for the predicted function and incorporated into the final version of the model (*i*ZM4_478).

**Table 1:** Comparison of *Z*. *mobilis* genome-scale metabolic models.

Model:	*i*ZM4_478	ZmoMBEL601	*i*ZM363	*i*ZM411	*i*EM439^b^
Reference:	(This Study)	[17]	[18]	[19]	[20]
Year of publication:	2020	2010	2011	2018	2016
**Number of reactions**^a^	747	591	739	648	755
**- Reactions w/ GPR**	612	498	414	507	593
**- Reactions w/o GPR**	135	93	325	141	162
**- Metabolic reactions w/o GPR**	19	64	182	89	106
**Number of genes**	478	353	363	360	439
**Number of metabolites**	616	579	600	602	658

Presented values are based on analysis performed with the model files included with the original publications to allow for a meaningful comparison between models and may not match values reported in these publications.

^*a*^ Reaction counts exclude biomass and exchange reactions. Metabolic reactions further exclude transport reactions across the cellular membranes.

^*b*^ *i*EM439 is a genome-scale metabolic model for ZM1.

## Results

### The genome-scale metabolic model of *Zymomonas mobilis ZM4*

Three genome-scale metabolic models of *Z*. *mobilis* strain ZM4 and one model of strain ZM1 have been previously published [17–20]; however, the previous ZM4 models are either not available in a simulation ready format, not charge balanced, or lack complete GPRs distinguishing between isozymes and subunits. Our newly developed genome-scale metabolic model of *Z*. *mobilis* ZM4 (*i*ZM4_478) contains 478 genes, 616 unique metabolites, and 747 mass- and charge-balanced metabolic and transport reactions, of which 612 have GPR associations (see S1 and S2 Models for SBML and Excel versions of the model). Out of the 135 reactions without GPR associations, 116 are transport reactions across the outer membrane with unknown porin specificity. This new model contains only 19 metabolic reactions lacking GPR associations, significantly fewer than previously developed metabolic models of *Z*. *mobilis* which ranged from 64 to 182 reactions lacking gene associations (Table 1).

### Comparison between model predictions and experimental data

**Fig 1:**
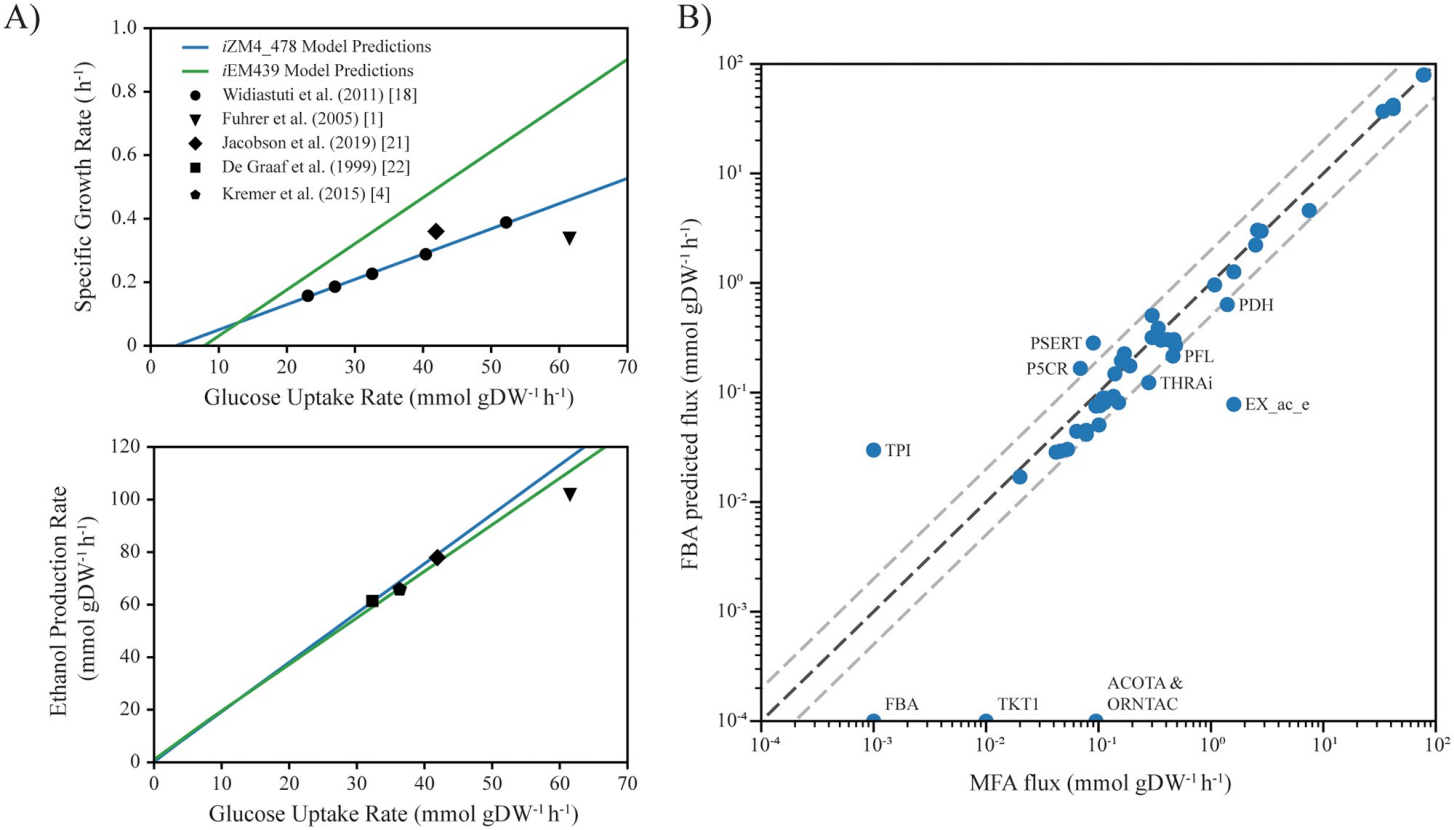
Model predictions for growth, ethanol and central metabolic fluxes. (A) Comparison between model predicted (solid lines) and reported experimental (data points) specific growth rates (top) and ethanol production rates (bottom) against glucose uptake rates for published anaerobic glucose minimal media experiments. Simulation ready models for *i*ZM363, *i*ZM411, and *i*ZmobMBEL601 were not available with their respective publications. (B) Plot of the predicted fluxes based on FBA versus the fluxes found via metabolic flux analysis in Jacobson et al. FBA was run for anaerobic growth in minimal media with a constraint preventing lactate production, forcing flux to ethanol as the primary fermentation product. Flux variability as determined by FVA at the optimal FBA growth rate is less than the size of the markers for each reaction. Grey dashed lines represent a 2-fold change in flux in either direction, reactions falling outside of these boundaries are labeled with their reaction IDs from *i*ZM4_478 (where ACOTA is acetylornithine transaminase, EX_ac_e is acetate exchange, FBA is fructose-bisphosphate aldolase, ORNTAC is ornithine transacetylase, P5CR is pyrroline-5-carboxylate reductase, PDH is pyruvate dehydrogenase, PFL is pyruvate formate lyase, PSERT is phosphoserine transaminase, THRAi is threonine aldolase, TKT1 is transketolase reaction involving sedoheptulose-7-phosphate, and TPI is the triose-phosphate isomerase). Reactions with zero flux in the FBA solution are plotted at 10^−4^ in the log space.

Flux balance analysis of *i*ZM4_478 accurately predicts specific growth rates and ethanol productivities. Multiple chemostat studies and ^13^C based metabolic flux analysis studies of *Z*. *mobilis* have been published with varying values in glucose uptake rates, specific growth rates, and ethanol productivities [1,4,18,21,22]. In order to compare model predictions across these uptake rates, we carried out FBA for glucose uptake rates ranging from 0 to 70 mmol/gDW/h. Predicted specific growth rates and ethanol production rates over this range are plotted alongside data points from previous experimental studies (Fig 1A). Additionally, *i*ZM4_478 was capable of predicting the growth rate and ethanol productivity from a study under nitrogen fixation conditions [4] (S1 Fig), a condition previous models were incapable of predicting accurately.

In addition to growth and fermentation rates, we compared the FBA predicted flux distribution, and carried out flux variability analysis (FVA) at the FBA optimal growth rate, against a recently published flux map derived from ^2^H and ^13^C metabolic flux analysis (MFA) (see Fig 1B and S1 Text) [21]. We generally found good correlation between the FBA predicted central metabolic fluxes and the experimentally derived fluxes, with a few outliers. At the optimal growth rate, the solution is nearly unique, with some flexibility in the utilization of NADH or NADPH in some few reactions. Predicted fluxes tended to underpredict flux towards biomass due to differences in specific growth rates at the specified glucose uptake rate (Fig 1A), along with key differences in fluxes of the pentose phosphate pathway, ornithine production, and excretion of acetate (see S1 Text for more detailed discussion of these results).

A phenotype microarray study of *Zymomonas mobilis* ZM4 identified a limited number of utilized substrates [24]. Only hexose sugars, glucose, fructose or the disaccharide sucrose, were consumed as primary carbon sources. Nitrogen sources were limited to ammonia, aspartate, asparagine, glutamate, glutamine, ethanolamine, glucuronamide, adenosine, parabanic acid, and some peptides. The model predicts growth on glucose, fructose, and sucrose as primary carbon sources and glutamine, ammonia, asparagine, and molecular nitrogen as nitrogen sources. Simulated growth with aspartate and glutamate as primary nitrogen sources requires the addition only of transport reactions for these metabolites. However, to simulate growth with ethanolamine, parabanic acid, or glucuronamide as priamry nitrogen sources requires both addition of transport and metabolic pathways. Since genes encoding pathways for utilizing these compounds as nitrogen sources are not known in *Z*. *mobilis* and we did not confirm their use in this study, reactions enabling growth for these nitrogen sources were not added to our model.

### Comparison of model predictions to transposon library data for growth in minimal media

During the development of our model, *in silico* predictions of gene essentiality were compared to the experimental results from individual experiments in previously published pooled Tn5 transposon mutant fitness experiments originally constructed to investigate chemical stresses from complex plant hydrolysates, and later extended to investigate mutant phenotypes [25,26]. While this dataset contains many experimental conditions carried out in a complex condition such as rich media, inclusion of inhibitory compounds, non-media environmental shifts (e.g., temperature), or motility assays, that are not modellable via genome-scale metabolic models, several experiments within the dataset can be modeled individually.

To investigate the completeness of the metabolic network and the predictive capability of our model, predictions under anaerobic minimal media conditions were compared against two replicate experiments, specifically Exp. 633 and Exp. 638 in the dataset. In order to compare to the model, the mutants in the experiments must be categorized as having either a growth phenotype or a non-growth phenotype. We selected a cutoff of -0.6 as the gene fitness score above which a mutant is considered to have a growth phenotype in the pooled mutant experiments. The fitness score cutoff influences the error rate between *in silico* model predictions and pooled phenotypes. A lower fitness score cutoff increases the number of false negatives (i.e., model predicts no growth, experimental data indicates growth), and a higher cutoff increases the number of false positives (i.e., model predicts growth, the experimental data indicates no growth). Further complicating the selection of a fitness score cutoff, the two experiments in the dataset may disagree regarding a given gene’s essentiality due to differences in fitness scores, giving rise to genes with an inconsistent pooled phenotype.

**Fig 2:**
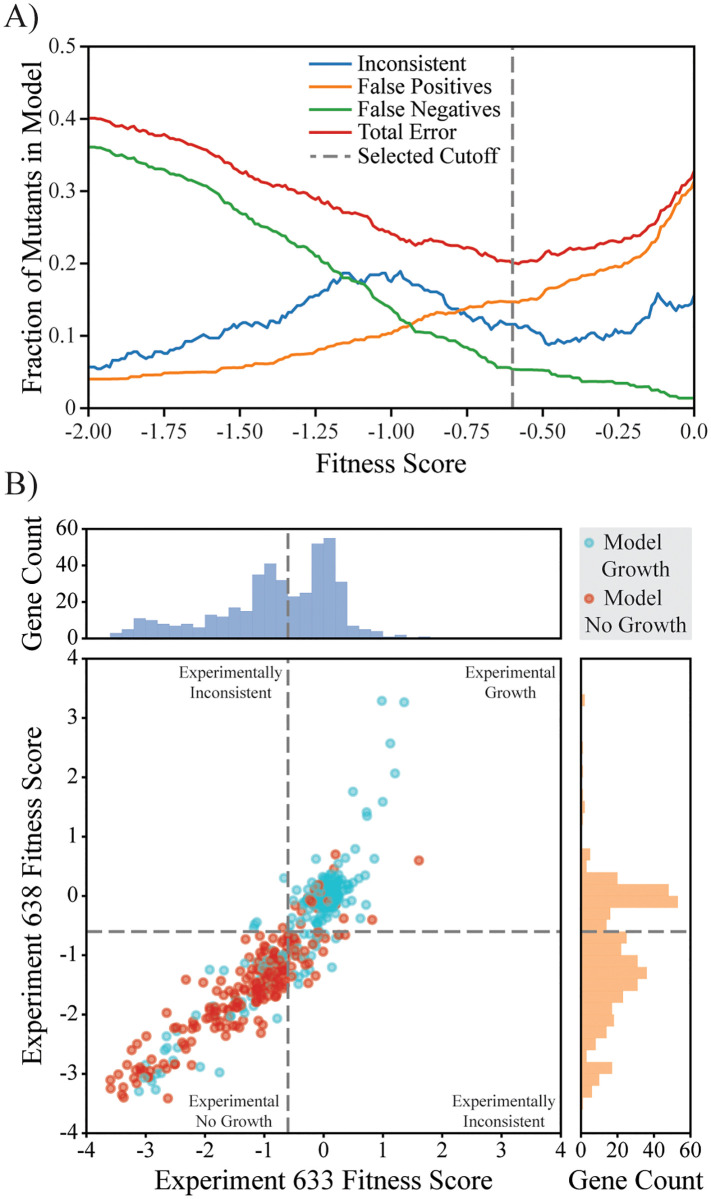
Comparison of model predictions to pooled fitness data. (A) Analysis of the effect of fitness score cutoff for growth phenotype classification of the pooled growth experiments. The fraction of genes with inconsistent growth phenotypes (i.e., above the cutoff in one experiment and below in the other) is shown in blue, the fraction of false positives (model predicts growth, but categorized as no growth experimentally, i.e., GNG mutants) in orange, the fraction of false negatives (model predicts no growth, but categorized as growing experimentally, NGG mutants) in green, and the total model prediction error (combination of false positive and negatives) is shown in red. The vertical dashed grey line represents the selected fitness score cutoff of -0.6 which minimizes the total error. (B) The fitness scores for genes included in *i*ZM4_478 for the two anaerobic glucose minimal media experiments (Exp. 633 and Exp. 638) are shown as a scatter plot and histograms. The fitness cutoff used for growth classification is shown as dashed grey lines. Genes in the scatter plot are colored based on model growth predictions, with cyan being genes predicted to be non-essential and red being genes predicted to be essential. Genes in the upper-left and lower-right quadrants of the scatter plot are genes where the growth phenotypes are inconsistent between the two experiments.

We selected a fitness score cutoff that minimized the total error between the model and the pooled mutant datasets thereby providing a higher confidence in identifying true mispredictions made by the model (Fig 2). With a cutoff of -0.6, transposon mutants in 380 genes included in the model exhibited consistent experimental mutant growth phenotypes in the anaerobic glucose minimal medium experiments. An additional 49 mutants had inconsistent experimental growth phenotypes (fitness score above -0.6 in one experiment and below -0.6 in the other), roughly half (24/49) of which were predicted by the model to be essential (Table 2). These 49 genes associated with inconsistent growth phenotypes were not considered further when comparing model predictions to the data. Under the selected cutoff, the final model predictions agree with the data for 81.58% (310/380) of the genes, with 13.16% (50/380) being false positives (GNG mutants, i.e., model predicts **G**rowth but experimental results indicate **N**o **G**rowth) and 5.26% (20/380) being false negatives (NGG mutants, i.e., model predicts **N**o **G**rowth but experimental results indicate **G**rowth). The relationship between model predictions and experimental growth phenotypes is statistically significant (chi-squared test statistic yields p < 0.001). There were 49 genes included in the model for which a mutant was not present in the Tn5 mutant collection, likely representing genes that are essential in the aerobic rich media in which the collection was made [25]. Roughly, two-thirds of these genes (33/49) were predicted to be essential by the model for growth in anaerobic glucose minimal media. A summary is shown in Table 2.

**Table 2:** Comparison of *in silico* predictions of single knockout mutants vs. pooled experimental results for anaerobic growth in minimal media.

		Experimental Results^a^			
		Growth	No growth	Inconsistent^b^	Unavailable^c^
**Model Predictions**	Growth	142 (GG)	50 (GNG)	25 (GI)	16
	No growth	20 (NGG)	167 (NGNG)	24 (NGI)	33

Abbreviations included in the table correspond to the abbreviations used in the text and are defined as the intersection of the categories (e.g., GG stands for model predicts Growth, experimental results indicate Growth).

^*a*^ Based on experimental results from Exp. 633 and Exp. 638 in Deutschbauer et al. [26].

^*b*^ The growth/no growth phenotypes for these mutants are different in the two experimental datasets.

^*c*^ The mutant was not available in the dataset.

In addition to the anaerobic minimal media condition, we modeled and similarly analyzed data from individual experiments using aerobic minimal media, alternative nitrogen sources (glutamine, glutamate, cysteine, nitrogen gas), and supplementation experiments with methionine or casamino acids. We found that the model performed similarly well at predicting growth/no growth phenotypes in these experiments, except for an experiment with no added nitrogen source. Except for that experiment, model predictions agreed with the fitness data for an average 77.2% of the genes, with a 14.3% false positive and 8.5% false negative rate. We found 26 genes were consistently classified as false positives (GNG) and 16 genes consistently classified as false negatives (NGG). A summary of these additional simulations is included in the supplemental S1 Text.

### Characterizing mispredicted Tn5 mutant isolates

Discrepancies between model predictions and experimental results can arise due to errors in the model, errors in the library or dataset, or as the result of the selected fitness cutoff used to assign experimental mutant growth phenotypes. During their analysis of the constructed transposon library in *Z*. *mobilis*, Skerker et al. noted that in some mutants PCR amplification of the mapped insertion region resulted in two bands, one corresponding to the wild-type gene and the other corresponding to the transposon disrupted gene (30). Therefore, to confirm that the differences between our draft model’s predictions and the dataset were true errors, we investigated 12 mutants from the collection. We selected five NGG mutants (two isolates of ZMO0113:Tn5, and single isolates of ZMO114::Tn5, ZMO0563::Tn5, ZMO0938::Tn5, and ZMO1307::Tn5), four GNG mutants (ZMO0962::Tn5, ZMO1494::Tn5, ZMO1488::Tn5, and ZMO1661::Tn5), two mutants predicted to grow but inconsistent experimentally (GI mutants ZMO1556::Tn5 and ZMO1598::Tn5), and one mutant the model and data agreed should grow (ZMO0172::Tn5, a GG mutant).

To confirm these mutants had transposons disrupting the gene to which the barcode was mapped, we amplified the mapped loci using PCR. Only five of the 12 (ZMO0114::Tn5, ZMO0172::Tn5, ZMO0938::Tn5, ZMO1307::Tn5, and one of the two isolates of ZMO0113::Tn5) mutants tested showed the expected product of the transposon disruptions of the mapped genes. An additional five mutants showed bands corresponding to both a disrupted and a wild-type copy of the gene, while gel imaging of the PCR products for the remaining two mutants revealed only the wild-type gene at the mapped loci. A summary of the PCR results is included with the strain list in Supplementary S2 Appendix.

For four of the five verified mutants with a pooled experimental “growth” phenotype, anaerobic growth experiments were performed in Hungate tubes with *Zymomonas* Minimal Medium Glucose (ZMMG). After one day, ZMO0938::Tn5 and ZMO1307::Tn5 reached an OD_600_ over 1.0, while ZMO0114::Tn5 and ZMO0172::Tn5 showed only weak growth. These latter two strains were diluted, transferred to fresh ZMMG medium, and incubated for an additional two days. ZMO0172::Tn5, a GG mutant in thiamine biosynthesis, grew with supplemented thiamine agreeing with both the model and experimental dataset. ZMO0114::Tn5, a NGG mutant involved in folate biosynthesis did not grow agreeing with our model prediction that this gene is essential, but not the dataset. It is possible that the ZMO0114::Tn5 mutant grew in the pooled fitness experiment by cross-feeding with other mutants, or using residual carryover from rich media precultures. ZMO0938::Tn5, a folate biosynthesis mutant, and ZMO1307::Tn5 a fumarase mutant both grew, agreeing with the experimental dataset, but not our initial model’s prediction that these genes are essential. The initial model’s ZMO1307 misprediction was caused by imbalanced flux through the fumarate node of the network. We found that due to five reactions that must occur in a fixed ratio due to the biomass equation and the lack of a complete TCA cycle in *Z*. *mobilis*, when the fumarase reaction was deleted from the model, no steady-state solution could be found (S2 Fig). Noting that the problem could be corrected via the excretion of fumarate *in silico*, we hypothesized and found that during growth ZMO1307::Tn5 excretes fumarate into the media. Inclusion of fumarate secretion in our model also resolved a NGG discrepancy for the malic enzyme mutant, ZMO1955::Tn5. The remaining discrepancy in folate biosynthesis (ZMO0938::Tn5) remains the same between the draft and final model and highlights that folate biosynthesis represent an area for further investigation and future improvements.

### Analyzing gene fitness correlations via flux coupling analysis

Flux coupling analysis was used to identify metabolic modules within our genome-scale model consisting of reactions that must carry flux simultaneously at variable (partially coupled) or fixed (fully coupled) ratios [32]. This analysis takes into account branch points in pathways that can lead to non-coupled reactions. With a full *in silico* media, allowing for the uptake or excretion of any metabolites with transport reactions included in the model, we identified 157 fully coupled and six partially coupled modules, containing between two and 19 reactions (see supplementary S1 Appendix for a complete list of modules). The corresponding set of genes, and their respective mutants in the collection, were identified for each module based on the GPR associations for these reactions. Mutants with isozymes were excluded from these sets.

Transposon mutants within the same metabolic module should result in similar phenotypic behavior in pooled experiments across different conditions, and the gene fitness scores between these mutants should be well correlated (e.g., mutants in the histidine biosynthesis pathway should all exhibit a histidine auxotrophy phenotype and have similar fitness scores in each experiment). The Pearson’s correlation for all pairs of genes’ fitness scores (cofitness) was calculated for all 492 experiments in the dataset. The mean cofitness was 0.07 for all genes and 0.15 for genes included in *i*ZM4_478. In order to determine if a given module showed a significant correlation in its gene’s fitness scores, we calculated threshold cofitness values representing the 95^th^ percentile of average cofitness scores of 100,000 sets of randomly sampled genes for a given module size (S1 Appendix). Thirty-two of the 39 modules containing three or more genes had average cofitness score above the corresponding threshold indicating genes in the module exhibited consistent mutant phenotypes. Reactions, genes, and associated pathways are shown in Table 3 for a subset of the metabolic modules, and in supplemental S1 Appendix for all modules. Modules with average cofitness values below the threshold may represent areas where our knowledge is incomplete and warrant further investigation, these modules are discussed in detail in supplementary S1 Text. Well correlating modules both validate our knowledge of *Z*. *mobilis* metabolism and can be applied to identify gene candidates for enzymes catalyzing reactions in a module lacking an annotated gene.

**Table 3:** Cofitness scores of select metabolic modules.

Module No.	Average Cofitness^a^	No. of Rxn	Coupled Reactions^b^	No. of Genes	Relevant Mutants^c^	Associated Pathways
**Modules with the highest average cofitness scores**					
M1	0.880	4	ALAS_f, HMBS_f, PPBNGS_f, UPP3S_f	4	ZMO1198, ZMO1879, ZMO1903	Porphyrinogen Biosynthesis
M2	0.859	3	CHORS_f, PSCVT_f, SHKK_f	3	ZMO0594, ZMO1693, ZMO1796	Chorismate biosynthesis
M3	0.846	4	ASPCT_f, *DHORTS_r*, OMPDC_f, ORPT_r	3	ZMO0587, ZMO0791, ZMO1707	Uridine biosynthesis
**Modules associated with histidine and cofactor biosynthesis**						
M10	0.746	10	ATPPRT_f, HISTD_f, **HISTP_f**, HSTPT_f, IG3PS_f, IGPDH_f, PRAMPC_f, PRATPP_f, PRMICI_f, SINK_his-L_f	9	ZMO0421, ZMO1178, ZMO1499, ZMO1500, ZMO1501, ZMO1502, ZMO1503, ZMO1550, ZMO1551	Histidine biosynthesis
M46	0.470	19	AMAOTr_f, AOXSr2_f, BTS5_f, DBTS_f, DM_AMOB_f, EGMEACPR_f, EPMEACPR_f, EX_meoh_e_f, MALCOAMT_f, MEOHtex_r, MEOHtrpp_r, *OGMEACPD_f*, OGMEACPR_f, OGMEACPS_f, OPMEACPD_f, OPMEACPR_f, OPMEACPS_f, **PMEACPE_f**, S2FE2SR_f	13	ZMO0094, ZMO0423, ZMO0425, ZMO0426, ZMO0427, ZMO1067, ZMO1146, ZMO1222, ZMO1278, ZMO1692, ZMO1915, ZMO1917, ZMO1918,	Biotin biosynthesis
**Modules with average cofitness scores below the significance threshold**					
M45	0.497	5	*E4PD_f*, OHPBAT_f, PDX5PS_f, **PERD_f**, SINK_pydx5p_f	3	ZMO1313, ZMO1684, ZMO1708	Pyridoxine biosynthesis
M51	0.346	8	EX_h2_e`_f, EX_n2_e_*r*, *FNOR_r*, H2tex_r, H2tpp_r, N2tex_f, N2tpp_f, NIT1b_f	3	ZMO1823, ZMO1824, ZMO1825	Nitrogen fixation
M52	0.332	5	CDPMEK_f, *DXPRIi_f*, MECDPDH5_f, MECDPS_f, *MEPCT_f*	4	ZMO0180, ZMO1128, ZMO1182, ZMO1851	Isoprenoid Precursor biosynthesis
M56	0.293	3	PGCD_f, PSERT_f, PSP_L_f	3	ZMO1137, ZMO1684, ZMO1685	Serine biosynthesis
M57	0.247	6	ADEt2rpp_r, ADEtex_r, HPN1_f, HPN2_f, **HPN3_f**, EX_ade_e_f	3	ZMO0873, ZMO0874, ZMO0969	Hopanoid biosynthesis
M60	0.119	11	ADCL_f, ADCS_f, AKP1_f, DHFS_f, *DHNPA2r_f*, DHPS2_f, EX_gcald_e_f, GCALDtex_f, GCALDtpp_f, GTPCI_f, *HPPK2_f*	7	ZMO0113, ZMO0114, ZMO0582, ZMO0938, ZMO1006, ZMO1229, ZMO1277	Folate biosynthesis
M63	-0.140	7	AMPMS2_f, DM_4CRSOL_f, ICYSDS_f, *PMPK_f*, **THZPSN3_f**, *TMPPP_f*, TYRL_f	3	ZMO0172, ZMO0738, ZMO1834	Thiamine biosynthesis

^*a*^ Module number shows relative position of module based on sorted average cofitness values.

^*b*^ Suffix "_f" represents forward component and "_r" represents reverse component of the reaction. Reactions italicized either had no available mutant in the collection, or had isozymes confounding analysis. Underlined reactions represent exchange or sink reactions missing GPRs. Bolded reactions represent reactions missing GPRs.

^*c*^ Mutants whose genes are associated with the reactions in the module excluding known isozymes and mutants that are absent from the Tn5 mutant collection.

### Identifying a missing gene in histidine biosynthesis

**Fig 3:**
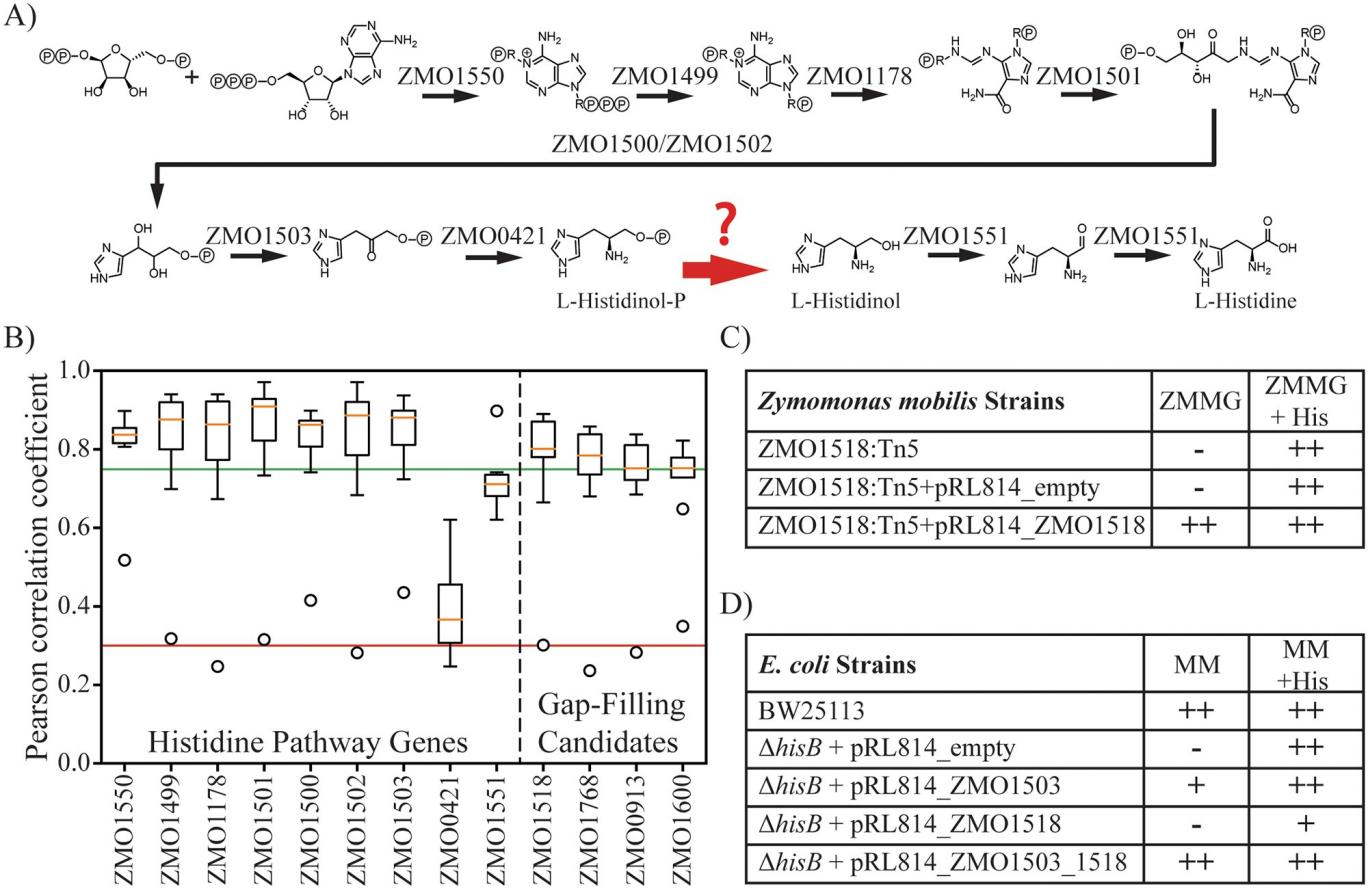
Identification of the histidinol-phosphatase gene. (A) An overview of the histidine biosynthesis pathway, converting 5-phosphoribosyl diphosphate (PRPP) to L-histidine. Note that ZMO1500 and ZMO1502 are subunits associated with the same reaction. Highlighted in red is the gap-filled histidinol-phosphatase reaction lacking an annotated gene. (B) Boxplot of the cofitness values for genes with known histidine biosynthesis genes. Cofitness of the genes in the histidine pathway with the eight genes of the histidine pathway are shown on the left. Candidate genes for the histidinol-phosphatase reaction (i.e., those with the highest average cofitness to the known genes) are shown on the right. The low cofitness outlier in all cases (except ZMO1551) corresponds to the cofitness with the ZMO0421 gene. (C) Growth experiments showed the ZMO1518::Tn5 mutant was a histidine auxotroph that could be rescued by complementation with ZMO1518 on a plasmid. Phenotypes are categorized as growth (++), weak growth (+), and no growth (-). (D) Growth experiments in *E*. *coli* Δ*hisB* demonstrate that ZMO1518 encodes a histidinol-phosphatase. Note that in *E*. *coli*, *hisB* has two functions catalyzing both the sixth and eighth steps in histidine biosynthesis, and therefore the *E*. *coli* Δ*hisB* knockout requires complementation with both ZMO1503 and ZMO1518.

During the draft model building process, we found that the gene responsible for the eighth step of histidine biosynthesis, histidinol-phosphatase, was missing from the genome annotation (Fig 3). This reaction must occur biologically since *Z*. *mobilis* ZM4 grows without histidine supplementation. In order to identify candidate histidinol-phosphatase encoding genes, we searched for additional genes in the dataset whose fitness were highly correlated with known histidine biosynthesis genes. With the exception of ZMO0421 where a transposon insertion had a polar effect on a downstream gene (discussed in supplemental S1 Text), the known genes in the histidine biosynthesis module (M10 in Table 3) were well correlated in the experimental datasets, with an average cofitness for the module of 0.75 (Fig 3). All genes were ranked by their average cofitness score with known genes in the pathway (S3 Fig), and the top genes were considered as potential candidates (Fig 3). The highest correlating gene ZMO1518 (mean r = 0.76) was annotated as an inositol-monophosphatase and was therefore considered a highly likely candidate. Other genes with high cofitness scores, ZMO1768 (mean r = 0.73), ZMO0913 (mean r = 0.72), and ZMO1600 (mean r = 0.71) were annotated as a diaminopimelate decarboxylase, branched-chain aminotransferase, and homoserine kinase, respectively.

The ZMO1518::Tn5 mutant and the other mutants in the histidine biosynthesis pathway were confirmed as histidine auxotrophs via growth experiments with and without histidine supplementation (S4 Fig). Complementation of the ZMO1518 gene on a plasmid rescued the ZMO1518::Tn5 mutant’s growth in un-supplemented minimal medium, indicating that the histidine auxotrophy was a result of the disrupted gene (Fig 3 and S4 Fig). To demonstrate that the ZMO1518 gene encodes a histidinol-phosphatase, we expressed ZMO1518 in a histidinol-phosphatase knockout strain of *E*. *coli*. In *E*. *coli*, the histidinol-phosphatase reaction is catalyzed by a multifunctional enzyme (HisB), which catalyzes both the sixth and eighth reaction steps in histidine biosynthesis. Expression of ZMO1503 (associated with the sixth step in *Z*. *mobilis*) and ZMO1518 rescued growth of an *E*. *coli* Δ*hisB* mutant in minimal medium lacking histidine (Fig 3 and S5 Fig). These complementation experiments demonstrate that ZMO1518 is responsible for catalyzing the histidinol-phosphatase reaction in *Z*. *mobilis*. After validating our candidate gene, we found that ZMO1518 was recently re-annotated as a histidinol-phosphatase based on computational inference in BioCyc [34] and our results provide the first experimental evidence confirming this prediction.

### Conditional anaerobic flux coupling and gene fitness correlations

During model construction, gap filling was required to allow for the anaerobic synthesis of ubiquinol and pyridoxal 5’-phosphate, as canonical steps in these pathways require molecular oxygen. We set out to investigate if genes responsible for alternative reactions that can function anaerobically could be identified through correlation within the dataset. We predict that O_2_-independent reactions should be essential in the anaerobic condition and should become coupled and therefore correlate well within the subset of experiments carried out anaerobically.

We carried out the same flux coupling and fitness correlation analysis except under anaerobic conditions, by constraining the oxygen uptake rate to zero, thereby simulating a rich anaerobic media. We found that nine of the previously identified metabolic modules gain additional condition-dependent fully coupled reactions. In six modules, these additional reactions become coupled as alternative aerobic reactions requiring molecular oxygen become infeasible. In the other three modules, alternative pathways require ubiquinone dependent oxidation reduction reactions coupled to the electron transport chain, which becomes blocked without oxygen as the terminal electron acceptor. Fitness correlation analysis similar to that described above for the full *in silico* media was conducted using the subset of 41 anaerobic experiments within the dataset, and modules identified via anaerobic flux coupling. The condition-dependent coupled reactions, genes, average cofitness scores for the anaerobic modules resulting from this analysis, and 95^th^ percentile cutoffs for randomly sampled gene sets within the anaerobic sub-dataset are presented in supplemental S1 Appendix.

We found that the modules with condition-dependent coupled reactions, did exhibit higher average cofitness scores in the anaerobic subset of the data. We hoped that by investigating the anaerobic cofitness of genes to the other genes in the ubiquinol (M11) or pyridoxal 5’-phosphate (M45) biosynthesis modules, we would be able to identify strong gene candidates for O_2_ independent reactions for these pathways. Unfortunately, no strong candidates were identified with this approach. While we were not successful in using this approach to identify additional genes, we did observe condition specific effects on the average cofitness of certain modules.

We found that module 8, representing the transport and phosphorylation of gluconate, has an average cofitness of 0.750 within the full dataset, but only 0.214 within the subset of anaerobic experiments. While *Z*. *mobilis* transports glucose via a facilitated diffusion process under most conditions, extracellular gluconate accumulation, the result of a periplasmic glucose dehydrogenase, has been described as a result of oxygen exposure [35]. The poor correlation of this module in the anaerobic case may be interpreted as these genes not being expressed or carrying minimal flux in the anaerobic state, agreeing with this previous observation.

### Identifying additional genes for reactions missing GPRs

We used our model-enabled gene search (MEGS) approach [33] to identify *Z*. *mobilis* genes likely responsible for other gap-filled reactions—chorismate-pyruvate lyase (encoded by *ubiC* in *E*. *coli*) and erythronate-4-phosphate dehydrogenase (encoded by *pdxB* in *E*. *coli*)—and to confirm the hypothesized pimeloyl-ACP methyl ester esterase (encoded by *bioH* in *E*. *coli*) (Table 4). MEGS was also applied to identify ZMO0201 as an isozyme of the ZMO0113 glutamine amidotransferase, and unsuccessfully to search for an isozyme of the ZMO1684 phosphoserine aminotransferase (supplemental S1 Text). Following the MEGS approach, first an *E*. *coli* host strain and selective medium pair were designed such that a reaction of interest (e.g., a gap-filled reaction) becomes essential in the *E*. *coli* host strain when grown in the paired selective medium. The corresponding gene in *Z*. *mobilis* was identified via a selection experiment after transformation into the host strain a plasmid-based library containing fragments of the *Z*. *mobilis* genome. Plasmids from the *Z*. *mobilis* library that rescue the *E*. *coli* host strain’s growth on the selective medium should contain *Z*. *mobilis* gene(s) responsible for the reaction of interest and were analyzed by DNA sequencing.

**Table 4:** Summary of identified genes.

*Z*. *mobilis* Gene	Experimental Activity	Identification Method	MEGS host *E*. *coli* Strain	Previous KEGG Annotation
ZMO0201	Glutamine amidotransferase of 4-amino-4-deoxychorismate synthase (isozyme)	MEGS	Δ*pabA*	Glutamine amidotransferase of anthranilate synthase
ZMO0563	Chorismate-pyruvate lyase	MEGS	Δ*ubiC*	Chorismate mutase
ZMO1008	Erythronate-4-phosphate dehydrogenase	MEGS	Δ*pdxB*	FAD linked oxidase domain protein
ZMO1518	Histidinol phosphatase	Bar-Seq Correlation	N/A	Inositol-monophosphatase
ZMO1916	Pimeloyl-ACP methyl ester esterase	MEGS	Δ*bioH*	Conserved Hypothetical Protein

*E*. *coli* knockout mutants Δ*ubiC*, Δ*pdxB*, and Δ*bioH* were used as MEGS host strains and paired selective media was MOPS minimal medium with 20 mM malate (Δ*ubiC*), MOPS minimal medium with 20 mM glucose (Δ*pdxB*), or M9 minimal medium with 20 mM glucose (Δ*bioH*), respectively. Plasmids that contained ZMO1008 or ZMO1916 complemented growth of the Δ*pdxB* and Δ*bioH E*. *coli* mutants, respectively. Therefore, ZMO1008 (annotated as a FAD linked oxidase domain protein) likely encodes an erythronate-4-phosphate dehydrogenase and ZMO1916 (annotated as a hypothetical protein) likely encodes a pimeloyl-ACP methyl ester esterase. ZMO0562 and ZMO0563 were found together on the plasmid complementing the growth of the Δ*ubiC E*. *coli* mutant. These two genes were cloned separately into the *E*. *coli* Δ*ubiC* strain and only ZMO0563 (annotated as chorismate mutase) complemented growth of the mutant, indicating that it is likely the enzyme responsible for chorismate-pyruvate lyase activity.

## Discussion

In this study, we analyzed a previously published pooled mutant fitness dataset using the framework of a newly developed genome-scale metabolic model of *Z*. *mobilis* ZM4 to improve the accuracy and gene assignments included in the model. Candidate genes for several gap-filled reactions were identified via correlations in the pooled fitness dataset or MEGS, and experimentally validated, allowing us to further improve the GPR assignments in our new genome-scale model *i*ZM4_478. Prior models of *Z*. *mobilis* contained large numbers of reactions without GPR associations. The model reported here has better gene coverage, with only 19 metabolic reactions lacking GPR associations in the final model (detailed in S1 Appendix).

The fitness score data from two experiments in the mutant fitness dataset, performed in anaerobic glucose minimal media, were compared to our model’s *in silico* predictions of gene essentiality. Disagreements between the model and dataset in this minimal media condition were especially useful for pointing out parts of the metabolic network that required attention and led to a correction in our draft model with the addition of fumarate secretion after confirming that ZMO1307::Tn5 was capable of growth in minimal media. However, not all mispredicted mutants were due to errors in the model. As noted by Skerker et al. and others who have worked with isolates from this *Z*. *mobilis* transposon library, some mutants carry both wild-type and transposon disrupted copies of the gene [25,26,36]. Indeed, we found several of the mutant isolates that we worked with presented both wild-type and disrupted gene bands after PCR. This observation has led to speculation that *Z*. *mobilis* may be polyploid [25,26] and recently Brenac et al. reported that *Z*. *mobilis* may carry more than 50 copies of its genome per cell [36]. Whatever the explanation, a mixed mutant genotype complicates the direct comparison between gene fitness scores from individual experiments and predictions made *in silico* by flux balance analysis. As a result, it is important to verify a strain’s phenotype in monoculture and genotype before making model changes based on discrepancies between experiments and model predictions.

Growth experiments with a subset of mutants confirmed two true errors in the model and revealed that in some cases cross-feeding or carry over of nutrients from rich media precultures may alter the expected phenotype in pooled experiments. ZMO0114::Tn5, a mutant in the folate biosynthesis module, initially grew weakly when transferred to minimal media. However, propagation of the strain to fresh media resulted in no growth indicating that nutrient cross-feeding or carryover may be supporting the strain’s growth in pooled experiments. ZMO0938::Tn5, another mutant in the folate biosynthesis module, represents a true misprediction made by our model. Furthermore, in our later analysis of metabolic modules we found that the folate biosynthesis module, M60, was poorly correlated. Together these suggest that the accuracy of the model’s representation, and thereby our understanding of how folate biosynthesis occurs in *Z*. *mobilis* is limited and an area deserving of further research.

In addition to analyzing individual experiments contained in the dataset, the correlation of fitness scores across the entire dataset provided a method for investigating pathways, or metabolic modules identified via flux coupling analysis of *i*ZM4_478. We expect mutants of genes in the same linear pathways to have the same phenotypic behavior in each experiment. Similarly, mutants for genes partially coupled through stoichiometric ratios should have similar phenotypic behavior as zero flux through one reaction implies zero flux through the other. Therefore, we posit that by using the Pearson correlations coefficient between pairs of genes across the entire dataset, we can mitigate the impact that mutants carrying both the wild-type and transposon disrupted gene may have during the analysis of the modules. With this approach, we found that by analyzing the pairwise cofitness of genes within the histidine module, we were able to successfully identify, and then experimentally confirm, the gene associated with histidinol-phosphatase, a reaction that had been added to the model during the gap-filling process.

We also investigated the condition-dependent correlation of fitness scores for the anaerobic subset of data. While we were unable to identify genes for the gapfilled reactions allowing for the anaerobic biosynthesis of cofactors, we did discover that condition-dependent coupling does occur within the dataset in the form of periplasmic glucose oxidation that has previously been observed after exposure to oxygen [35]. We were unable to investigate other condition-dependent correlations, as other identified conditions (e.g., alternative nitrogen sources) within the dataset lacked a statistically powerful number of individual experiments for determining cofitness scores. Condition-dependent flux coupling analysis may be an avenue worth considering in the design, collection and applications of similar datasets for genome-scale metabolic module refinement.

**Fig 4:**
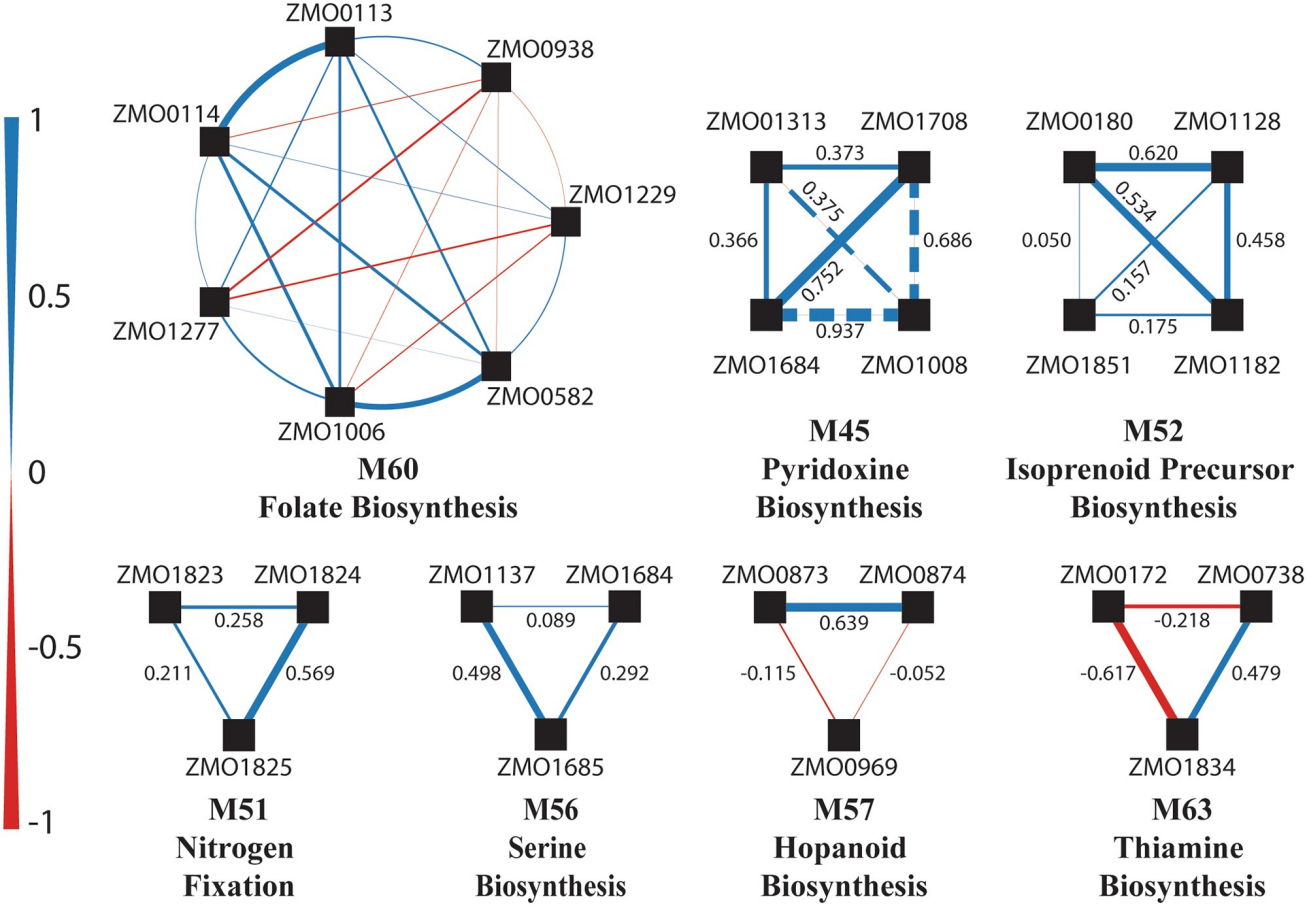
Pairwise cofitness in poorly correlating modules. The seven poorly correlating modules are represented as node and edge plots. Each node (black square) is a gene within the module, while each edge between nodes represents the cofitness for that pair of genes. Edges in blue represent positive cofitness and edges in red negative cofitness. Edge thickness correspond to the value of cofitness. These pairwise cofitness values are shown for smaller modules. In Module 45, the gene ZMO1008 was identified via MEGS during model development and is shown here with dashed lines.

While most of the modules in *Z*. *mobilis* are well correlated across the entire mutant fitness dataset, several are not. Investigation of pairwise cofitness of genes in these modules (Fig 4, S1 Text) revealed that in several cases, a single poorly correlated gene lowered the average cofitness. We found several cases in which this may be explained by either the genetic context, or as an artifact of the pooled experiments. In the case of the ZMO0421::Tn5 mutant, polar effects on a downstream gene caused an expected histidine auxotrophy, but an unexpected tyrosine auxotrophy (S1 Text, S6 Fig). This unexpected phenotype likely led to the low cofitness scores we observed between ZMO0421 and the other genes in the histidine pathway. Growth experiments with ZMO0114::Tn5 and ZMO0172::Tn5 mutants suggests that media carryover or cross-feeding may obscure true growth phenotypes within the pooled experiments. In other cases, such as the nitrogen fixation module (M51) or thiamine biosynthesis module (M63), the media in the majority of experimental conditions in the pooled dataset contained the relevant nutrients making the genes in the module non-essential, which may explain their low correlation. While many of these poorly correlating genes could be explained, others may be the result of errors within the mutants in the collection or may represent true knowledge gaps in our understanding of *Z*. *mobilis* metabolism.

In conjunction with the analysis performed using the published dataset, we applied MEGS [33] to experimentally investigate several more gap-filled reactions and poorly correlated modules and identified three genes (ZMO1008, and ZMO1916, and ZMO0563) for reactions without GPRs in the initial model. Further supporting our identification as an erythronate-4-phosphate dehydrogenase, ZMO1008 has a high cofitness score with other mutants in the gene module encompassing pyridoxal 5’-phosphate synthesis, ranking third following the same procedure used to identify candidate genes for the histidine pathway (S3 Fig). ZMO1916, the identified pimeloyl-ACP methyl ester esterase, on the other hand would have been difficult to identify via this approach as it ranked 131^st^ when ordered by average cofitness to the rest of the pathway (S3 Fig). The chorismate-pyruvate lyase reaction did not belong to an identified metabolic module, but MEGS identified ZMO0563, annotated as a chorismate mutase, a gene that if overexpressed overcomes the loss of chorismate-pyruvate lyase in *E*. *coli*. Our results provide experimental evidence for the annotation of the *Z*. *mobilis* genes responsible for chorismate-pyruvate lyase and erythronate-4-phosphatase. Furthermore, the results support a previous inference for the gene encoding pimeloyl ACP methyl ester esterase made in the original study of the pooled fitness dataset [26], where ZMO1916 was observed to have high cofitness scores with biotin synthase (ZMO0094, r = 0.95) and dethiobiotin synthase (ZMO1915, r = 0.87).

Our results show how pooled fitness Tn-Seq or Bar-seq datasets can be helpful in the development and validation of a metabolic model, and how these datasets can be leveraged in the identification of genes responsible for gap-filled reactions. The presence of mis-mapped barcodes, unexpected polar effects, or mutants possessing both wild-type and transposon disrupted genes in mutant libraries is not unexpected, as the size of these collections makes characterizing each individual mutant prohibitive. Even with these experimental caveats, these high-throughput datasets still provide extremely valuable insight into the phenotype of most knockouts. The metabolic model for *Z*. *mobilis*, *i*ZM4_478, that we have employed, and improved via the analysis of the pooled mutant dataset we analyzed will be a useful tool for future studies and engineering efforts in this organism. Through this combination of computational and experimental approaches we identified and verified functions of four genes in *Z*. *mobilis* metabolism, which close knowledge gaps in histidine, biotin, ubiquinone, and pyridoxine biosynthesis. It is noteworthy that mixed genotype mutants have not been commonly observed in the construction of previous barcoded transposon libraries [25], and therefore the methods of analysis with genome-scale models we have conducted here are likely to be even more straightforward in organisms with similar datasets.

## Materials and methods

### *Z*. *mobilis* ZM4 metabolic reconstruction

*i*ZM4_478 was constructed with extensive manual curation using the *E*. *coli* model *i*JO1366 [37] as a template and source for gap-filling reactions using SMILEY [38]. The biomass and biomass component reactions were reconstructed from multiple sources and details can be found in supplemental S2 Model. The growth-associated maintenance (GAM) energy and non-growth associated maintenance (NGAM) energy, the additional energy requirement for cellular growth or maintenance functions for cell survival expressed and modeled in terms of ATP equivalents, were estimated to be 75.37 mmol ATP/gDW and 3.71 mmol ATP/gDW/h, respectively where gDW stands for gram dry weight. These parameters were fit to reported rates from buffered batch culture experiments [18]. Various resources and databases were used to support the reconstruction process, including NCBI [39], KEGG [40], BioCyc [34], BiGG [41], BRENDA [42], MicrobesOnline [43], TransportDB [44], and Memote [45]. Additional details can be found in the Supplemental S1 Text.

### Model-predicted growth, ethanol production, central metabolic fluxes, and growth phenotypes

Flux balance analysis (FBA) of *i*ZM4_478 and *i*EM439 was conducted over a range of glucose uptake rates ranging from 0 to 70 mmol/gDW/h for the anaerobic glucose minimal media, and anaerobic nitrogen fixing glucose minimal media conditions. Predicted specific growth rates and ethanol production rates were evaluated at each glucose uptake point. Simulated lactate production was constrained to zero flux. The *i*ZM4_478 flux distribution predicted by FBA at a glucose uptake rate of 41.9 mmol/gDW/h, was compared to the MFA determined fluxes. For lumped biosynthesis reactions in the MFA fit, the flux of a single representative flux from the linear pathway was compared. The ATPS4rpp reaction in *i*EM439 was constrained to a flux of -4 mmol/gDW/h for all simulations with this model, as utilized in the original manuscript [20].

Gene essentiality was predicted using FBA and a gene was considered essential if the maximum flux through the biomass reaction was predicted by FBA to be zero. In all simulations, the lower bounds on the exchange fluxes for biotin, thiamine, and all components found in *Zymomonas* Minimal Medium Glucose (ZMMG) were set to -1000 mmol/gDW/h allowing for uptake of these metabolites in the model. Glucose and oxygen exchange flux lower bounds were set to -61.5 and 0 mmol/gDW/h, respectively [1]. The upper bound on all exchange fluxes was 1000 mmol/gDW/h so that any extracellular compound may be secreted.

### Flux coupling analysis

The Flux Coupling Finder [32] was used to identify fully coupled and partially coupled reactions in the *Z*. *mobilis* model. In our analysis, all exchange fluxes were allowed to take positive or negative values and individual sink reactions for each biomass component and terminal products of complete metabolic pathways were added so that smaller modules of reactions coupled to individual biomass components (rather than aggregate biomass) could be found.

### Average cofitness cutoff based on gene module size

Cofitness was calculated as the Pearson’s correlation between the fitness scores for two genes across all 492 experiments for the full *in silico* modules, and across the 41 anaerobic experiments for the anaerobic condition-dependent modules. For a given set of genes, the average cofitness was calculated as the average of the cofitness scores between all pairwise combinations of genes in the set. To determine a significance cutoff for the average cofitness for a gene module of size k, we first sampled k genes randomly from the set of 423 metabolic genes whose mutants were included in the pooled fitness datasets, calculated the cofitness between each pairwise combination, and then calculated the average of k choose two pairwise cofitness scores. We repeated the random sampling of k genes 100,000 times and chose the average cofitness at the 95^th^ percentile as the cutoff. Similar analysis of the 41 anaerobic experiments was performed to determine 95^th^ percentile cutoffs for the anaerobic condition-dependent modules.

### Strain construction

*E*. *coli* BW25113 was obtained from *E*. *coli* genetic stock center and single knockout *E*. *coli* strains were obtained from the Keio collection (Open Biosystems) [46]. Strains with multiple gene deletions were constructed through sequential removal of the kanamycin resistance gene (kan) using the temperature sensitive pCP20 plasmid as described previously [47], prior to incorporation of additional deletions using P1 transduction [48] with single knockout mutants as donor strains. *Z*. *mobilis* ZM4 was obtained from ATCC (ATCC 31821). *Z*. *mobilis* ZM4 Tn5 mutants were obtained from the mutant collection generated by Skerker et al. [25] and used in the pooled fitness datasets reported by Deutschbauer et al. [26]. Strains used in this study are listed in supplemental S2 Appendix.

### Plasmid construction

Gibson cloning [49] was used to generate all plasmids expressing *Z*. *mobilis* genes in *E*. *coli* and *Z*. *mobilis*. The RBS sequence in front of ZMO1518 in pRL814_ZMO1503_ZMO1518 was designed using the Salis RBS Calculator [50] to have a similar translation rate as ZMO1503. The ZMO0421 and ZMO0420 genes in pRL814_ZMO0421_ZMO0420 were amplified from the genome with their natural spacing and sequences conserved. All plasmid inserts were confirmed via sequencing. Plasmids used in this work and primers used to construct them are listed in supplemental S2 Appendix.

### Media and cultivation

Unless otherwise noted, *E*. *coli* strains were grown at 37°C with shaking and *Z*. *mobilis* strains were grown at 30°C without shaking. *E*. *coli* strains were grown aerobically on LB agar plates, in LB broth, 2 g/L glucose M9 minimal medium (MM), or MM supplemented with 0.25 g/L aspartate, 0.2 g/L leucine, 0.15 g/L isoleucine and 0.15 g/L valine (MM++). *Z*. *mobilis* strains were grown in *Zymomonas* Rich Medium Glucose (ZRMG), *Zymomonas* Minimal Medium Glucose (ZMMG), *Zymomonas* Minimal Medium Glucose with reduced iron sulfate (ZMMG_0.25Fe), ZRMG agar plates, or ZRMG agar plates supplemented with 0.33 g/L each of histidine, tyrosine, and phenylalanine (ZRMG++ agar). When necessary, kanamycin or spectinomycin were added at final concentrations of 50 μg/mL for *E*. *coli* and 100 μg/mL for *Z*. *mobilis*. Media recipes can be found in supplemental S2 Appendix.

### Plate reader growth experiments

Strains were streaked onto the appropriate rich medium agar plates (LB or ZMRG) with the necessary antibiotics. Single colonies were precultured aerobically overnight in 3 mL rich medium with the appropriate antibiotics, centrifuged, washed once in minimal medium, and inoculated to a starting optical density of ~0.01 at 600 nm (OD_600_). All plate reader growth experiments were performed in triplicate in 96-well microplates at 200 μL volume. The OD_600_ of the *E*. *coli* cultures was measured by an Infinite M200 plate reader (Tecan) shaking at 37°C for 24 hours. For *Z*. *mobilis*, the OD_595_ was measured by an Infinite F500 plate reader (Tecan) placed inside an anaerobic chamber shaking at 31.5°C for 48 hours.

### Validation of the growth phenotypes Tn5 mutant isolates

*Z*. *mobilis* mutants selected for testing based on incorrect growth predictions were taken from frozen stocks and grown up in 3 mL ZRMG with kanamycin overnight before streaking on ZRMG agar plates with kanamycin. Primer pairs for each tested mutant isolate were designed with primers approximately 500 bp upstream and downstream of the mapped insertion site, such that the PCR product would include the inserted transposon if present. Independent PCR reactions were performed using three colonies from each plate. Mutant growth experiments were performed in duplicate. Strains were inoculated to a starting OD_600_ of ~0.02 in 5 mL of ZMMG_0.25Fe with kanamycin in Hungate tubes purged with filtered nitrogen gas, and grown for 24 hours. Mutants with poor growth were diluted, transferred to fresh media, and incubated for another 48 hours.

### Liquid chromatography-mass spectrometry of extracellular fumarate

Biological triplicates of ZMO1307::Tn5 grown in ZMMG were washed and inoculated into fresh ZMMG to a starting OD_600_ = 0.02 and grown to a final OD_600_ = 1.0. Supernatant samples were filtered through a 0.22 μm filter and diluted 100-fold in HPLC grade water. A fumarate standard curve was constructed in HPLC grade water to determine sample concentrations. Samples and standards were analyzed with a Vanquish UPLC coupled to a Q Exactive orbitrap high-resolution mass spectrometer (ThermoScientific) equipped with electrospray ionization operating in negative-ion mode. Chromatography was carried out on a 2.1 x 100 mm ACQUITY UHPLC BEH C_18_ column with 1.7 μm particle size (Waters) at 25°C. Solvent A was 97:3 water:methanol with 10 mM tributylamine adjusted to pH 8.2 with 10 mM acetic acid and solvent B was 100% methanol. The separation run time was 25 minutes following the protocol: 0 min, 5% B; 2.5 min, 5% B; 17 min, 95% B; 19.5 min, 95% B; 20 min, 5% B; 25 min 5% B. Full MS-SIM (single ion monitoring) parameters include scanning between 70 and 1000m/z, automatic control gain target of 1e6, maximum injection time of 40 ms, with a resolution of 140,000 full width at half maximum. Data analysis performed using the MAVEN software package with fumarate identified by retention time matched to the standard curve. Fumarate excretion was simulated in MATLAB 2017a as growth associated product formation based on the excretion rate determined by FBA, and integrated to OD_600_ = 1.0. OD_600_ values were converted to gDW based on a published conversion factor of 0.41 gDW OD_600_^-1^ L^-1^ [21]

### MEGS genomic libraries and growth selections

Growth coupled recipient *E*. *coli* strains and paired media sets were designed using FBA with the *E*. *coli i*JO1366 model [37] as described previously [33]. The genomic DNA library of *Z*. *mobilis* was prepared using fragments of extracted genomic DNA generated through sonication and ligated into the HinCII multiple cloning site of the pZE21MCS1 vector [33]. The average insert size of the library was ~1.4 kbp, with a titer of 210,000 colony forming units (CFUs).

## Supporting information

S1 Text Additional notes on model reconstruction, investigation of the ZMO0421::Tn5 mutant, and discussion of poorly correlating modules. (DOCX)

S1 Model *i*ZM4_478 model in SBML format. (XML)

S2 Model *i*ZM4_478 model in readable Excel format. (XLSX)

S1 Appendix Flux coupling analysis module average cofitness and cofitness thresholds, and summary of gap-filled reactions. (XLSX)

S2 Appendix Strain, plasmid, primer, and media lists. (XLSX)

S1 Fig Model predictions of growth and fermentative productivity under nitrogen fixation conditions. Comparison of model predicted (solid lines) and reported experimental (data points) specific growth rates (top) and ethanol production rates (bottom) against glucose uptake rates for published nitrogen fixation experiments conducted in anaerobic glucose minimal media experiments. Simulation ready models for *i*ZM363, *i*ZM411, and *i*ZmobMBEL601 were not available with their respective publications.
(TIF)

S2 Fig Fumarate fluxes in the ZMO1307::Tn5 mutant. (A) Diagram of reactions included in the model producing or consuming fumarate. For simplicity, fluxes shown were calculated with an *in silico* growth rate of 1 hr^-1^. Due to the composition of the biomass equation, several reactions producing fumarate from aspartic acid, or using fumarate as an electron acceptor (shown in red) are constrained to a fixed ratio with the biomass reaction since their products lead to ATP, GTP, UTP, CTP, NAD or arginine synthesis. Only fumarase (ν_FUM_) and fumarate transport reactions (ν_FUM1tpp_, ν_FUMtex_) are unconstrained and may carry the balance of flux (0.6474 mmol/gDW/hr) necessary to satisfy the steady state assumption around the fumarate (or fumaric acid) node. (B) Bar graph of extracellular fumarate concentration of ZMO1307::Tn5 culture at an OD_600_ = 1.0, model predicted concentration based shown in light blue, experimentally measured supernatant concentration shown in dark blue, error bar represents one standard deviation of triplicate data.
(TIF)

S3 Fig Cofitness analysis histidine, pyridoxine, and biotin biosynthesis modules. Boxplots of cofitness values of individual genes with the module genes and bar graphs showing genes ordered by average cofitness scores to module genes for modules 8, 42, and 46 (A, B, and C respectively). Cofitness of the genes in the module with the other genes in the module are shown in the left of the boxplot, candidate genes or genes identified via MEGS are shown to the right of the plot. Orange bars in the bargraph represent the module genes, blue bars the candidate genes included in the left panel, and green bars genes identified via MEGS that do not overlap with candidate genes. Horizontal green lines represent the average cofitness score of the known genes (0.746, 0.497, and 0.466 for panels A, B, and C respectively) and the red lines represent the 95^th^ percentile module cutoffs. ZMO1008, the gene identified via MEGS experiments, was identified as the gene with the third highest average cofitness score. ZMO1916, identified as the pimeloyl-ACP methyl ester esterase via MEGS experiments is shown on the far right of the boxplot and highlighted via a green bar in the bargraph.
(TIF)

S4 Fig Growth curves of histidine biosynthesis pathway Tn5 mutants and ZMO1518::Tn5 mutants. Growth of the wild-type and each tested mutant, or mutant with plasmid complement, shown in minimal media (ZMMG) (blue), and in minimal media with histidine supplementation (orange) over 48 hours. The solid line represents the average (n = 3) optical density at 595nm (OD595) over time, and the shaded band indicates one standard deviation.
(TIF)

S5 Fig Δ*hisB E*. *coli* growth curves for ZMO1518 verification. Growth curves of Δ*hisB E*. *coli* strain carrying an empty plasmid (blue), pRL814_ZMO1503 (orange), pRL814_ZMO1518 (green), and pRL814_ZMOM1503_ZMO1518 (red) in minimal media without supplementation. The solid line represents the average (n = 3) optical density at 600 nm (OD_600_) over time, and the shaded band indicates one standard deviation.
(TIF)

S6 Fig Overview of ZMO0421::Tn5 analysis. (A) Genomic region containing ZMO0421 and ZMO0420, which are in a single operon. (B) Partial pathways for tyrosine and histidine biosynthesis, showing the steps catalyzed by ZMO0421 and ZMO0420. (C) Summary of growth phenotypes of a *ΔaspC ΔtyrB ΔilvE* triple *E*. *coli* knockout with and without plasmid complementation of ZMO0421 grown in minimal media with no supplementation, supplementation with tyrosine, phenylalanine, or both. (D) Summary of the growth phenotypes for the ZMO0421::Tn5 mutant harboring different plasmids in minimal media with different supplementations. Phenotypes are categorized as growth (++), weak growth (+), and no growth (-).
(TIF)
