# Supplementary material for: Model-driven analysis of mutant fitness experiments improves genome-scale metabolic models of Zymomonas mobilis ZM4
Source: PLoS Comput Biol. 2020 Aug 17;16(8):e1008137. doi: 10.1371/journal.pcbi.1008137 (PMC7451989; doi:10.1371/journal.pcbi.1008137)
Supplement: S1 Text — (DOCX) [file pcbi.1008137.s001.docx]

Model-driven analysis of mutant fitness experiments improves genome-scale metabolic models of *Zymomonas mobilis* ZM4

Wai Kit Ong^1,3¶^, Dylan K. Courtney^1,3¶^, Shu Pan^1,3^, Ramon Bonela Andrade^1^, Patricia J. Kiley^2,3^, Brian F. Pfleger^1,3^, Jennifer L. Reed^1,3*^

^1^Department of Chemical and Biological Engineering, University of Wisconsin – Madison, Madison, WI, USA

^2^Department of Biomolecular Chemistry, University of Wisconsin – Madison, Madison, WI, USA

^3^DOE Great Lakes Bioenergy Research Center, Univ. of Wisconsin-Madison

*Corresponding author

Email: [Jennifer.Reed@wisc.edu](mailto:Jennifer.Reed@wisc.edu) (JR)

^¶^These authors contributed equally to this work.

**S1 Text**

***i*ZM4_478 model reconstruction - additional notes**

The *E. coli* model *i*JO1366 was used as a template for the manual reconstruction, copying reactions to the initial draft when orthologs to *E. coli* genes were found in *Z. mobilis* ZM4. We defined genes as being orthologs if they were the best reciprocal hits in the KEGG Sequence Similarity Database (SSDB) and were assigned the same KEGG orthology (KO) identifiers. Gap-filling was conducted with additional reactions from *i*JO1366 to allow for growth under glucose minimal media conditions following SMILEY [1]. Genes were identified manually for most of these gap-filled reactions based on the genome annotation by Yang et al. [2]. Alternative pathways and their associated *Z. mobilis* genes were added to the model to replace gap-filled reactions that could not be associated with any *Z. mobilis* genes. Additional metabolic genes identified in literature (and their associated reactions) were added to further complete the model. Finally, transport and exchange reactions were added to the model if those metabolites were found to be secreted or consumed experimentally.

**Comparison of *i*ZM4_478 flux balance analysis results to metabolic flux analysis flux maps.**

In order to determine the accuracy of the model’s predictive capacity for intracellular flux distribution we chose to compare it to the recently published metabolic flux analysis (MFA) flux model for *Z. mobilis* ZM4, that was fit using both ^13^C and ^2^H labeling [3]. Flux balance analysis (FBA) was used to predict the flux distribution based on the structure of the genome-scale metabolic model. Flux variability analysis (FVA) was carried out with the growth rate constrained to rate in the FBA solution to determine the uniqueness of the solution. At the maximum growth rate determined by FBA, the solution is almost unique. There is a small degree of flexibility between NADH and NADPH utilizing enzymes. Because the MFA flux model was generated using lumped reactions for the production of amino acids and other biomass precursors, we selected a single corresponding representative reaction from the genome-scale metabolic model for each reaction in the MFA model.

In several cases, the stoichiometric model contains additional routes for biosynthesis of amino acids, or fermentative products that the best fit MFA model does not have. We found that the genome scale metabolic model allows for production of ethanol or lactate as the final fermentative product, resulting in equivalent predicted growth rates. Because, *Z. mobilis* possesses an annotated lactate dehydrogenase, we included the reaction in the genome-scale metabolic model; however, ethanol is the primary fermentative end-product experimentally and is the primary product in the MFA model [3,4]. Therefore, we applied a constraint on the lactate dehydrogenase for the remainder of our comparisons to the MFA model. In addition, the MFA model also predicts a low flux (0.22 mmol gDW^-1^ h^-1^) excretion of acetate (reaction ID: EX_ac_e), that FBA does not. While many reactions correlate well (correlation coefficient = 0.49), there are some notable exceptions where the difference in flux is greater than 2-fold.

Four reactions in the MFA model carry no flux in the FBA results. Fructose-bisphosphate aldolase (reaction ID: FBA), does not carry flux in the genome-scale metabolic model because phosphofructokinase activity is not included, as there is no annotated gene corresponding to this reaction. In the development of the MFA model the authors found that inclusion of this reaction at extremely low flux (-0.001 mmol gDW^-1^ h^-1^) led to a statistically better fit. The transketolase reaction from ribose 5-phosphate and xylulose 5-phosphate to glyceraldehyde 3-phosphate and sedoheptulose 7-phosphate (reaction ID: TKT1) cannot carry flux in the model as sedoheptulose 7-phosphate is a dead-end metabolite. *Z. mobilis* has no annotated transaldolase, and no genetic evidence for other reactions using sedoheptulose 7-phosphate. This reaction was able to carry flux in the MFA model due to a demand associated with the defined biomass equation used in that study. Finally, the genome scale metabolic model produces ornithine for arginine biosynthesis from proline through the reverse reaction of ornithine cyclodeaminase. The MFA model instead produces ornithine from glutamate through N2-acetyl-L-ornithine. By going through proline FBA gains a net 0.001 gDW h^-1^ increase in the biomass growth rate compared to the path from glutamate. Relaxing the flux variability biomass growth rate constraint to 99% of the optimum growth rate found by FBA results in flexibility between these two pathways within the model (data not shown). Notably, forcing flux through the glutamate pathway in the analysis (data not shown) results in much better correlation of the ornithine transacetylase and acetylornithine transaminase reactions (reaction IDs: ORNTAC and ACOTA respectively), as well as correcting the overprediction of pyrroline-5-carboxylate reductase (reaction ID: P5CR) flux for proline biosynthesis.

In addition to the overprediction of flux towards proline, the FBA results also over predict flux through the triose-phosphate isomerase (reaction ID: TPI) and towards serine from 3-phosphoglycerate (representative reaction ID: PSERT). Relaxing the flux variability growth rate constraint allows for significant variability in the TPI reaction The genome scale metabolic model contains triacylglycerols in the biomass equation, while the MFA model does not. The necessary glycerol-3-phosphate are produced from dihydroxyacetone phosphate, the product of the triose-phosphate isomerase reaction, in FBA. Higher predicted flux through serine for the production of glycine also leads to the under-prediction of the threonine aldolase (reaction ID: THRAi) the route for glycine biosynthesis in the MFA model.

Finally, we do observe an under-prediction of fluxes directed towards amino acid and biomass biosynthesis (PDH and PFL reactions, and other low values not specifically denoted in S6 Fig) from the FBA results. We suspect this is a result of fitting the ATP maintenance values to the chemostat dataset presented by Widiastuti et al., in which the biomass yield on glucose was less than found by Jacobson et al [3,5]. At the glucose uptake rate of -41.9 mmol gDW^-1^ h^-1^ our model predicts a growth rate of 0.301 gDW h^-1^, instead of the 0.360 gDW h^-1^ observed by Jacobson et al. We would expect then, at the same glucose flux, for less flux to be directed towards amino acids and biomass precursors in the FBA results compared to the MFA model.

**Additional Analysis of Individual Experiments**

We have identified an additional 12 experiments in defined media from the larger dataset that lend themselves to flux balance analysis. Growth/no growth predictions for these experimental conditions were performed. Each experiment listed below was analyzed individually and the fitness cutoff set to minimize the model error as conducted for the main manuscript. Asterisks indicate that an exchange or transport reaction was necessary to add to the model to facilitate the simulation.

| **Bar-Seq Experiment** | **GG** | **NGNG** | **GNG** | **NGG** |
| --- | --- | --- | --- | --- |
| Aerobic Minimal Media – Exp 73 | 163 | 179 | 56 | 31 |
| Aerobic Minimal Media – Exp 281 | 152 | 185 | 67 | 25 |
| Aerobic Minimal Media – Exp 636 | 159 | 179 | 60 | 31 |
| Glutamine as Nitrogen Source – Exp 282 | 145 | 176 | 73 | 35 |
| Glutamate as Nitrogen Source – Exp 283* | 155 | 172 | 67 | 35 |
| Cysteine as Nitrogen Source – Exp 284* | 162 | 164 | 63 | 40 |
| No Nitrogen Source – Exp 641 | 189 | 40 | 24 | 176 |
| No Nitrogen Source; Plate Assay – Exp 642 | 153 | 186 | 60 | 30 |
| Methionine Supplementation 40ug/ml – Exp 76* | 160 | 179 | 62 | 28 |
| Methionine Supplementation 80ug/ml – Exp 77* | 163 | 176 | 59 | 31 |
| CAS amino acid Supplementation 0.2% - Exp 74 | 163 | 156 | 56 | 54 |
| CAS amino acid Supplementation 0.4% - Exp 75 | 165 | 149 | 54 | 61 |

With the exception of experiment 641 with no nitrogen source, we see that the results of the analysis for each modelable condition is similar to what we found for the anaerobic minimal media condition (142 GG, 167 NGNG, 50 GNG, 20 NGG). It was unclear from the experiment descriptions if the nitrogen source and supplementation experiments were carried out aerobically or anaerobically. We have assumed aerobically as all anaerobic experiments were labeled as such. It is noteworthy that *Z. mobilis* is capable of fixing nitrogen only under strict anaerobic conditions, as such we would expect little to no growth under aerobic conditions with no nitrogen source. While notes of growth for each condition were not reported, we do find that the distribution of gene fitness scores is more normally distributed around zero compared to other experiments as one might expect from a pooled transposon experiment with limited or no growth (data not shown). In contrast the distribution for the plate assay with no nitrogen source (experiment 642) displays a distribution more like other experiments, which leads us to wonder if anaerobic conditions might be achieved within colonies of *Z. mobilis* growing on plates aerobically.

Except for the no nitrogen source experiment (Exp 641), we found relatively good consistency between the replicate experiments. We found that many of the incorrectly predicted genes by the model were also mispredicted in many of these modelable experimental conditions. Sixteen genes were found to be predicted to be essential by the model but found to be non-essential (NGG) in each of the pooled experiments, excluding the no nitrogen assay. Similarly, 26 genes predicted to be non-essential by the model but found to be essential in the pooled experiment (GNG) were common to all experiments excluding the no nitrogen assays.

Within these common mispredicted sets, we identified some gene clustering based on functionality. Within the NGG genes, we observe issues with folate and one carbon metabolism, as well as flavodoxin, and transporters. In the GNG set of genes, multiple genes associated with glutathione/glutaredoxin and heme precursor synthesis were present, as were complexes for pyruvate dehydrogenase and lipid transport. Multiple GNG genes present are not essential *in silico* because of the inclusion of annotated isozymes, that may not be truly functional.

**Investigation of the ZMO0421::Tn5 mutant**

With the observed poor correlation of the ZMO0421 gene with other genes in the histidine biosynthesis pathway, we hypothesized that ZMO0421, an aminotransferase, may play a role in other biosynthesis pathway(s), or that the insertion of a transposon in ZMO0421 disrupted expression of ZMO0420, a gene downstream in the same operon (Fig. S8A). ZMO0420 is annotated as an arogenate dehydrogenase, responsible for making the immediate precursor to tyrosine (Fig. S8B). ZMO0420 was predicted by our model, and experimentally found to be essential for anaerobic minimal media growth in the pooled fitness experiments.

To evaluate if ZMO0421 is involved in aminotransferase activity in tyrosine or phenylalanine biosynthesis, we constructed a triple transaminase knockout (Δ*aspC*Δ*tyrB ilvE*::*kan*) *E. coli* mutant, and verified that it was a branched chain amino acid, aspartate, phenylalanine and tyrosine auxotroph. Complementation of the ZMO0421 gene on a plasmid rescued the *E. coli* triple gene deletion mutant’s growth in minimal medium supplemented with branched chain amino acids and aspartate (MM++), demonstrating that ZMO0421 exhibits phenylalanine and tyrosine aminotransferase activities in *E. coli* (Fig. S8C and S9).

Supplementation of minimal media with tyrosine and histidine (but not phenylalanine) restored robust growth of the ZMO0421::Tn5 mutant; however, plasmid based expression of ZMO0421 did not rescue the mutant’s growth in minimal medium, implying a possible polar effect. The same mutant transformed with ZMO0420 was able to grow without the addition of tyrosine, and exhibited only a histidine auxotrophy. Finally, complementation of both ZMO0420 and ZMO0421 fully restored the ability of the mutant to grow in minimal media (Fig. S8D and S10).

Together these experiments suggest that the transposon insertion in ZMO0421 had a polar effect disrupting the expression of ZMO0420, which is essential for tyrosine biosynthesis. Additionally, they demonstrate that ZMO0421 is responsible for aminotransferase activity in histidine biosynthesis, and can catalyze aminotransferase activity in the phenylalanine and tyrosine biosynthesis pathways, but the latter activities are also catalyzed by other gene products in *Z. mobilis*.

**Discussion for each flux coupling analysis module with poor experimental correlation**

The results of the analysis of mutant cofitness in metabolic modules presented in Figure 3 in the main text point towards the need to improve our understanding of various cofactor and prosthetic group biosynthesis reactions in *Z. mobilis*. Module correlation may be poor for various reasons and we discuss observations for each of the six poorly correlated modules below.

Module 45 (M45), consists of four metabolic reactions, a sink reaction and three relevant, available genes involved in the biosynthesis of pyridoxine. ZMO0177, the gene associated with erythrose 4-phosphate dehydrogenase was not available in the mutant dataset. The GPR for erythronate 4-phosphate dehydrogenase was unknown prior to identification as ZMO1008 via our MEGS analysis. Inclusion of ZMO1008 in the analysis of this module increases the average cofitness score from 0.497 to 0.581, above the 95^th^ percentile cutoff for modules with four mutants. However, the initial poor average cofitness in this module was the result of a single mutant being poorly correlated to other mutants in the pathway. ZMO1313, annotated as 4-hydroxythreonine-4-phosphate dehydrogenase and associated with pyridoxine 5’-phophate synthase has a cofitness of 0.375, 0.366, and 0.373 with ZMO1008, ZMO1684, and ZMO1708, respectively. The ZMO1313::Tn5 mutant was classified as NGI (model predicts no growth but experimentally inconsistent mutant) for the anaerobic glucose minimal media experiments. Furthermore, PCR analysis of one of the only two mutants in the mutant collection revealed that the barcode associated with this mutant was mismapped, or the heterozygous mutant had reverted to primarily or entirely the wild-type gene. It is likely that this contributed significantly to the poor correlation observed in the pooled data set.

Module 51 (M51) contains only two metabolic reactions, and six transport or exchange reactions. Further complicating the analysis of this module is ZMO1753, the gene associated with the ferredoxin-NADP reductase reaction is not available in the mutant dataset. Thus, only genes ZMO1823, ZMO1824, and ZMO1825, all components of the nitrogenase (NIT1b) reaction, are included in the module. These genes are poorly correlated across all experiments; however, these genes were only essential in experiments without a provided nitrogen source (2 of 492 experiments).

Module 52 (M52) includes five reactions associated with isoprenoid precursor biosynthesis through the methyl-erithyritol phosphate pathway. A mutant of ZMO1150, 1-deoxy-D-xylulose reductoisomerase, was not present in the pooled library, and isozyme for the 2-C-methyl-D-erythritol 4-phosphate cytidylyltransferase, ZMO0353, was excluded from the analysis. ZMO1851, annotated as a flavodoxin, was poorly correlated with the remaining three mutants with cofitness scores of 0.050, 0.157 and 0.175 for ZMO0180, ZMO1128, and ZMO1182, respectively, and was mispredicted by the model as a NGG mutant in anaerobic glucose minimal media conditions. It is possible that the association of this flavodoxin with several other reactions led to poor correlation, a different flavoprotein may be associated with this reaction, or mutants in the collection may have gene duplication events or barcode mismapping.

Module 56 (M56) consists of three genes for three metabolic reactions converting 3-phosphoglycerate into serine. All three mutants in this module were found to be essential in the anaerobic glucose minimal media experiments, but were incorrectly predicted by the model to be non-essential as the model includes an alternate pathway to make serine from glycine through the glycine hydroxymethyltransferase reaction. ZMO1684 correlates poorly with the other two genes in this module, lowering the average cofitness score. Due to this poor correlation, we applied MEGS to search for a possible isozyme for the phosphoserine aminotransferase encoded by ZMO1684; however, only ZMO1684 was found in the plasmids rescuing growth of an *E. coli* Δ*serC* mutant. It remains unclear why these serine biosynthesis mutants show poor cofitness and should be investigated further.

Module 57 (M57) contains three metabolic reactions, and three transport or exchange reactions. Three relevant genes, corresponding to two of the hopanoid biosynthesis genes, and one putative transporter for adenine. These reactions are responsible for the conversion of hopene to tetrahydroxybacteriohopane that is incorporated into the cellular membrane. These reactions are not well understood, and follow the schema presented by Belin et al. [6]. Homologs for HpnH and HpnG were identified within the chromosome, the enzyme responsible for the conversion of ribosylhopane to Tetrahydroxybacteriohopane remains unknown. Based on the reaction schema an adenine is removed from adenosylhopane by HpnG. This results in the coupling of these reactions to the export of adenine as salvage pathways for adenine do not exist within the model and are not annotated in databases such as KEGG. However, the putative transporter for adenine has a weak negative correlation to HpnG and HpnH, resulting in the poor correlation observed in this module. Further research into these steps of hopanoid biosynthesis in *Z. mobilis* may be necessary to determine if the proposed schema is representative in this organism, and adenine salvage likely occurs or if differences in the hopanoid synthesis pathway exist.

Module 60 (M60) includes eight metabolic reactions and three transport or exchange reactions. Seven relevant mutants, corresponding to six of the metabolic reactions were represented in the dataset. Mutants of ZMO1059, encoding for dihydroneopterin aldolase, and ZMO1647, encoding for 6-hydroxymethyl-dihydropterin pyrophosphokinase, were not in the mutant collection. In M56, most of the available mutants have poor cofitness scores and some of the growth phenotypes do not match model predictions. As discussed previously, we found that one of the isolates of ZMO0113::Tn5 had wild-type and transposon disrupted copies of the gene, while another isolate had a homozygous transposon disrupted genotype. Furthermore, when testing the growth of ZMO0114::Tn5 isolate in anaerobic minimal media conditions, we found that media carryover was responsible for the initial growth phenotype. The glutamine amidotransferase component of the 4-amino-4-deoxychorismate synthase reaction, encoded by ZMO0113, has a pooled growth phenotype that was incorrectly predicted by the model as no growth, indicating a possible isozyme for ZMO0113 might exist. We used the MEGS approach to search for alternative isozymes for ZMO0113 and ZMO0114. *E. coli* ∆*pabA* and ∆*pabB* strains were used as a host strain to search for ZMO0113 and ZMO0114 isozymes in aerobic glucose M9 minimal medium. Genomic library plasmids containing either ZMO0113 or ZMO0201 rescued growth of the *E. coli* ∆*pabA* mutant while the ∆*pabB* was only rescued by plasmids containing ZMO0114. ZMO0201 is annotated as the glutamine amidotransferase of anthranilate synthase in KEGG. According to the KEGG annotation, ZMO0201 and ZMO0113 share the same substrates (chorismate and glutamine) and both are glutamine amidotransferases. The updated NCBI annotation for ZMO0201 indicates it has dual functions as aminodeoxychorismate / anthranilate synthase component II.

Module 63 (M63) contains six metabolic reactions and a demand reaction. The ZMO1003 mutant, annotated as the phosphomethylpyrimidine kinase, was not available in the Tn5 transposon mutant collection dataset. Furthermore, isozymes ZMO0332 and ZMO1425 were excluded from the analysis. Of the remaining genes, ZMO0172 was found to be weakly and negatively correlated with the other two mutants in the module (ZMO0738 and ZMO1834). A possible explanation for the low cofitness scores in this module is that the reactions were not necessary in most conditions as thiamine was present in the media for most of the pooled experiments.

**Supplementary References**

1. Reed JL, Patel TR, Chen KH, Joyce AR, Applebee MK, Herring CD, et al. Systems approach to refining genome annotation. Proc Natl Acad Sci. 2006;103(46):17480–4.

2. Yang S, Pappas KM, Hauser LJ, Land ML, Chen GL, Hurst GB, et al. Improved genome annotation for *Zymomonas mobilis*. Nat Biotechnol. 2009;27(10):893–4.

3. Jacobson TB, Adamczyk PA, Stevenson DM, Regner M, Ralph J, Reed JL, et al. 2H and 13C metabolic flux analysis elucidates in vivo thermodynamics of the ED pathway in Zymomonas mobilis. Metab Eng. 2019;54(March):301–16.

4. Fuhrer T, Fischer E, Sauer U. Experimental identification and quantification of glucose metabolism in seven bacterial species. J Bacteriol. 2005;187(5):1581–90.

5. Widiastuti H, Kim JY, Selvarasu S, Karimi IA, Kim H, Seo JS, et al. Genome-scale modeling and in silico analysis of ethanologenic bacteria *Zymomonas mobilis*. Biotechnol Bioeng. 2011;108(3):655–65.

6. Belin BJ, Busset N, Giraud E, Molinaro A, Silipo A, Newman DiK. Hopanoid lipids: From membranes to plant-bacteria interactions. Nat Rev Microbiol. 2018;16(5):304–15.
