# Supplementary figures and images for: Model-driven analysis of mutant fitness experiments improves genome-scale metabolic models of Zymomonas mobilis ZM4
Source: PLoS Comput Biol. 2020 Aug 17;16(8):e1008137. doi: 10.1371/journal.pcbi.1008137 (PMC7451989; doi:10.1371/journal.pcbi.1008137)

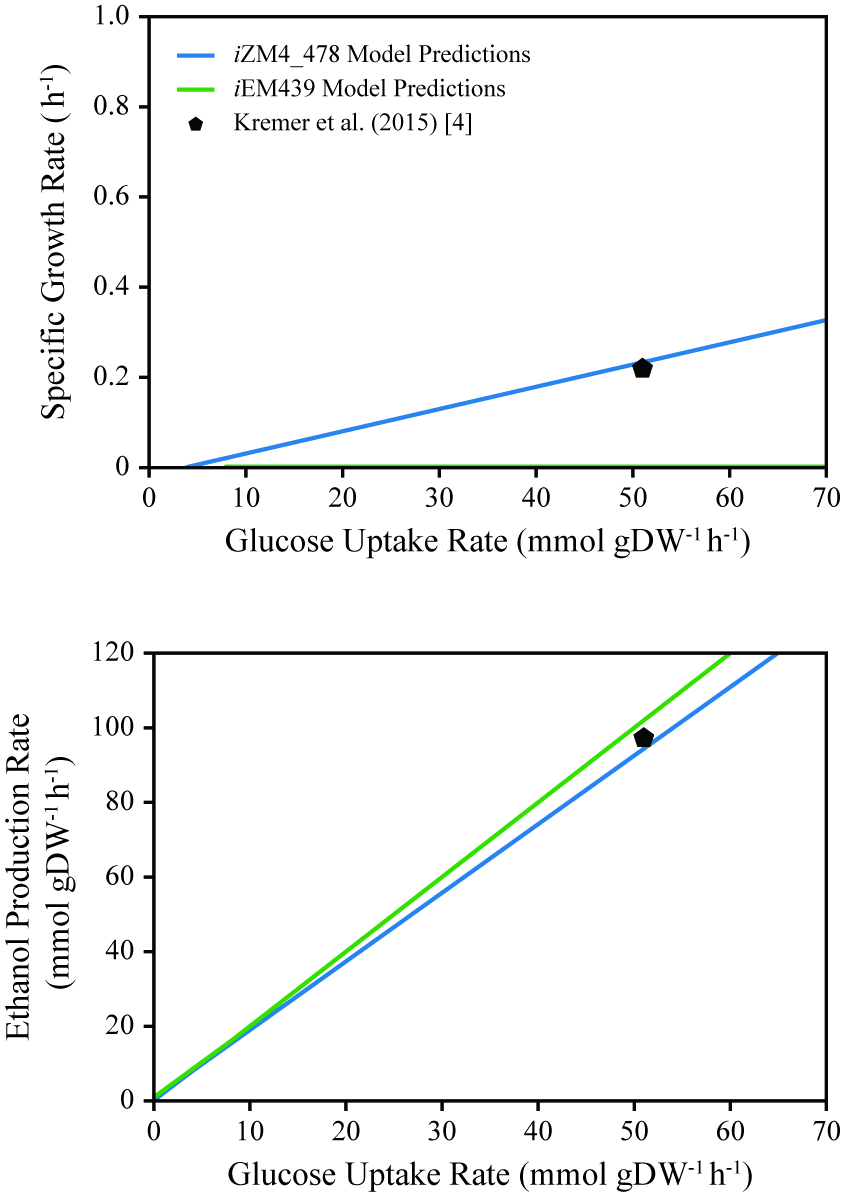

Supplement: S1 Fig — Comparison of model predicted (solid lines) and reported experimental (data points) specific growth rates (top) and ethanol production rates (bottom) against glucose uptake rates for published nitrogen fixation experiments conducted in anaerobic glucose minimal media experiments. Simulation ready models for iZM363, iZM411, and iZmobMBEL601 were not available with their respective publications. (TIF) [file pcbi.1008137.s006.tif]

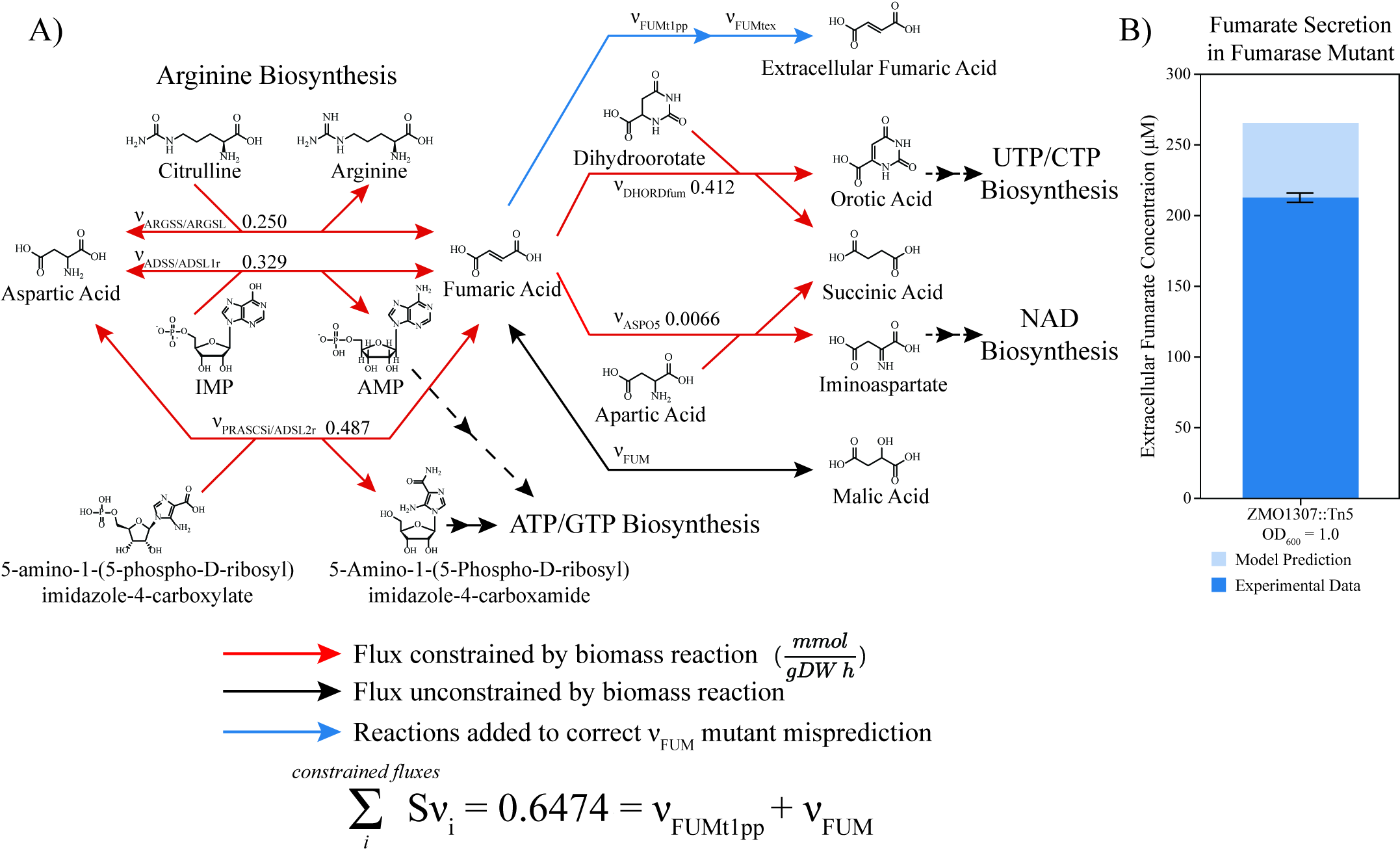

Supplement: S2 Fig — (A) Diagram of reactions included in the model producing or consuming fumarate. For simplicity, fluxes shown were calculated with an in silico growth rate of 1 hr-1. Due to the composition of the biomass equation, several reactions producing fumarate from aspartic acid, or using fumarate as an electron acceptor (shown in red) are constrained to a fixed ratio with the biomass reaction since their products lead to ATP, GTP, UTP, CTP, NAD or arginine synthesis. Only fumarase (νFUM) and fumarate transport reactions (νFUM1tpp, νFUMtex) are unconstrained and may carry the balance of flux (0.6474 mmol/gDW/hr) necessary to satisfy the steady state assumption around the fumarate (or fumaric acid) node. (B) Bar graph of extracellular fumarate concentration of ZMO1307::Tn5 culture at an OD600 = 1.0, model predicted concentration based shown in light blue, experimentally measured supernatant concentration shown in dark blue, error bar represents one standard deviation of triplicate data. (TIF) [file pcbi.1008137.s007.tif]

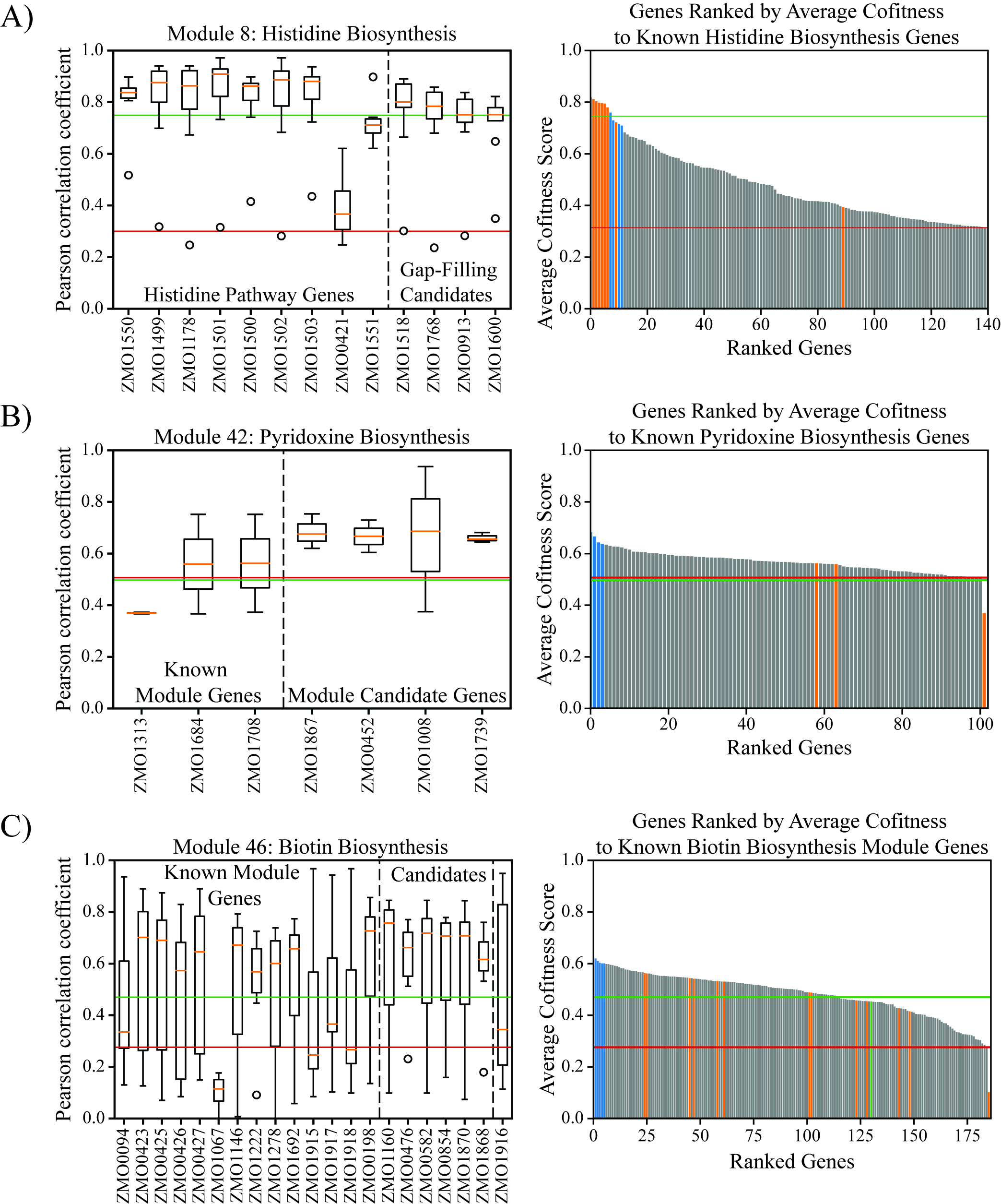

Supplement: S3 Fig — Boxplots of cofitness values of individual genes with the module genes and bar graphs showing genes ordered by average cofitness scores to module genes for modules 8, 42, and 46 (A, B, and C respectively). Cofitness of the genes in the module with the other genes in the module are shown in the left of the boxplot, candidate genes or genes identified via MEGS are shown to the right of the plot. Orange bars in the bargraph represent the module genes, blue bars the candidate genes included in the left panel, and green bars genes identified via MEGS that do not overlap with candidate genes. Horizontal green lines represent the average cofitness score of the known genes (0.746, 0.497, and 0.466 for panels A, B, and C respectively) and the red lines represent the 95th percentile module cutoffs. ZMO1008, the gene identified via MEGS experiments, was identified as the gene with the third highest average cofitness score. ZMO1916, identified as the pimeloyl-ACP methyl ester esterase via MEGS experiments is shown on the far right of the boxplot and highlighted via a green bar in the bargraph. (TIF) [file pcbi.1008137.s008.tif]

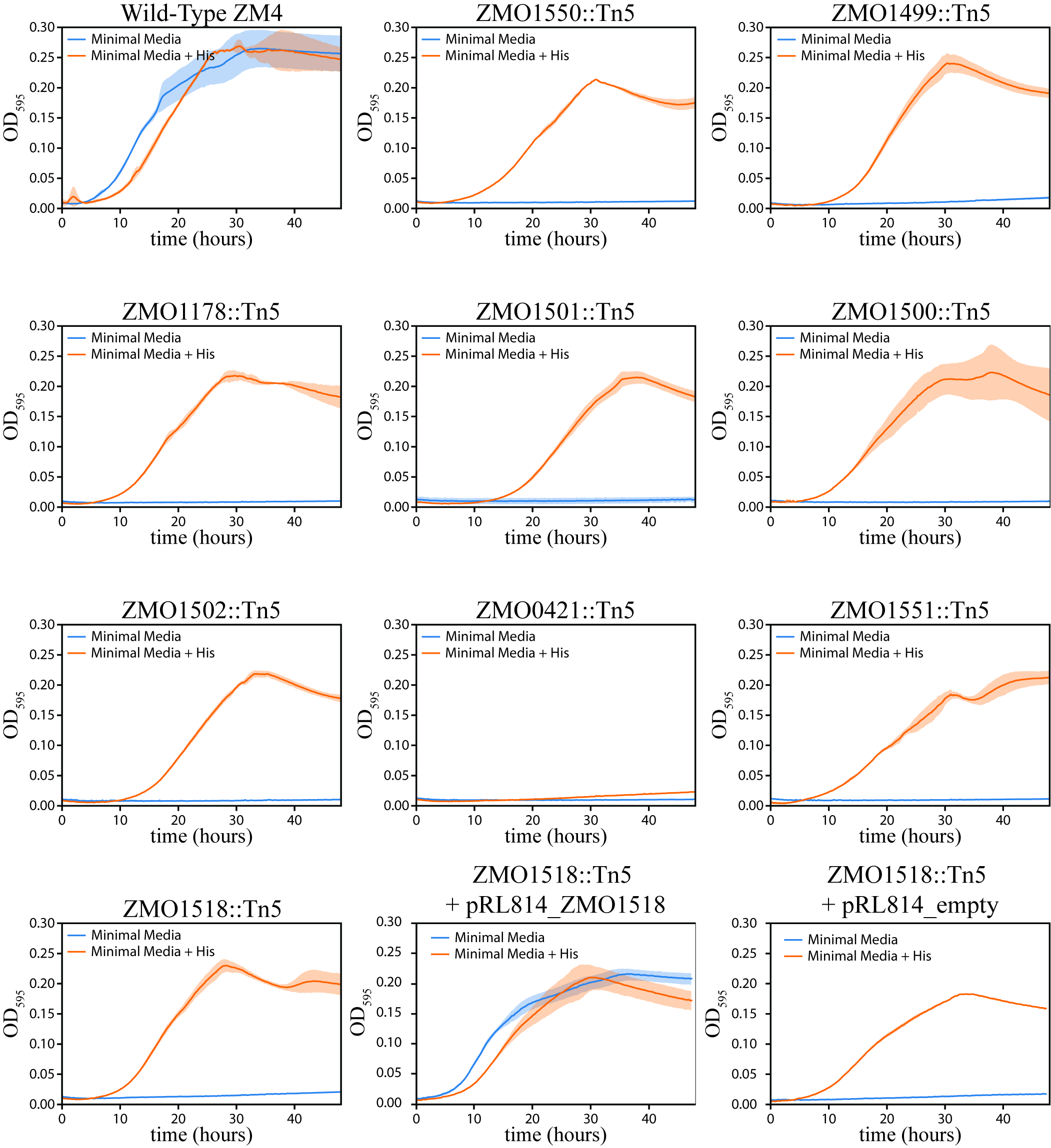

Supplement: S4 Fig — Growth of the wild-type and each tested mutant, or mutant with plasmid complement, shown in minimal media (ZMMG) (blue), and in minimal media with histidine supplementation (orange) over 48 hours. The solid line represents the average (n = 3) optical density at 595nm (OD595) over time, and the shaded band indicates one standard deviation. (TIF) [file pcbi.1008137.s009.tif]

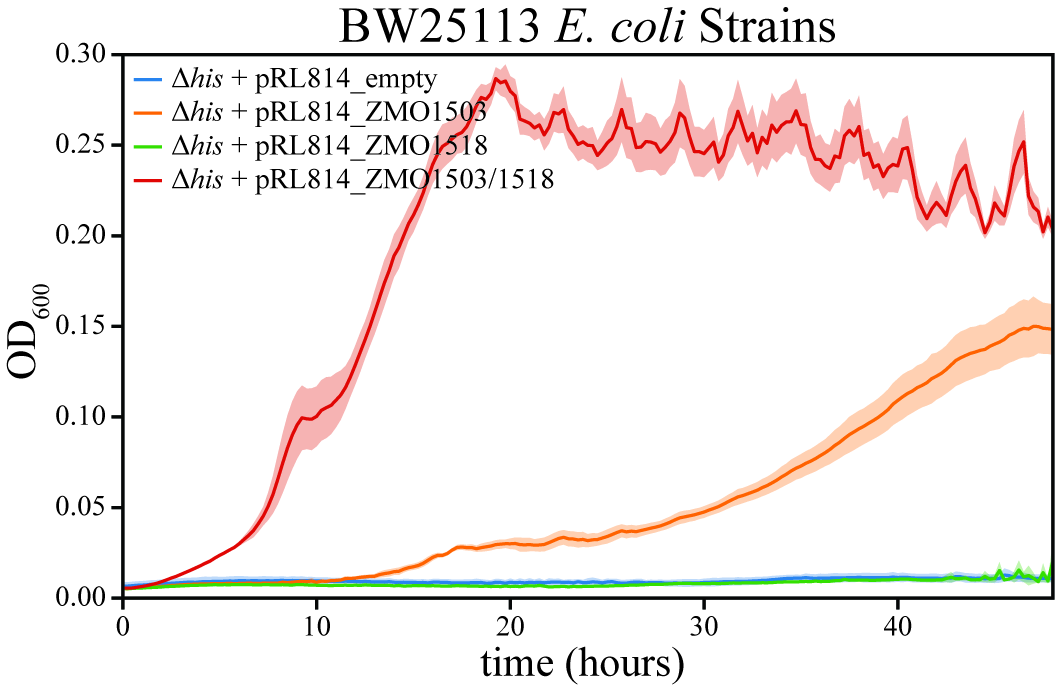

Supplement: S5 Fig — Growth curves of ΔhisB E. coli strain carrying an empty plasmid (blue), pRL814_ZMO1503 (orange), pRL814_ZMO1518 (green), and pRL814_ZMOM1503_ZMO1518 (red) in minimal media without supplementation. The solid line represents the average (n = 3) optical density at 600 nm (OD600) over time, and the shaded band indicates one standard deviation. (TIF) [file pcbi.1008137.s010.tif]

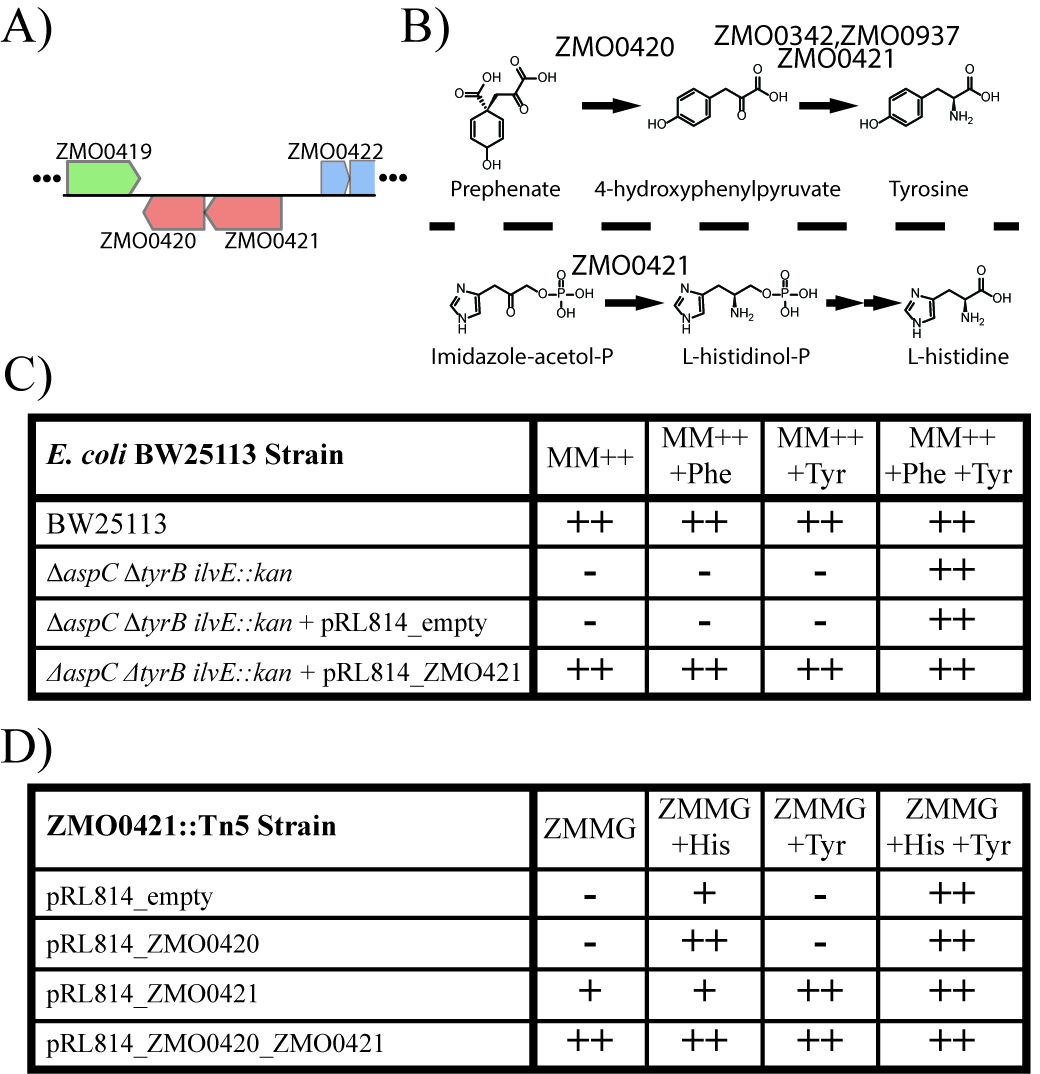

Supplement: S6 Fig — (A) Genomic region containing ZMO0421 and ZMO0420, which are in a single operon. (B) Partial pathways for tyrosine and histidine biosynthesis, showing the steps catalyzed by ZMO0421 and ZMO0420. (C) Summary of growth phenotypes of a ΔaspC ΔtyrB ΔilvE triple E. coli knockout with and without plasmid complementation of ZMO0421 grown in minimal media with no supplementation, supplementation with tyrosine, phenylalanine, or both. (D) Summary of the growth phenotypes for the ZMO0421::Tn5 mutant harboring different plasmids in minimal media with different supplementations. Phenotypes are categorized as growth (++), weak growth (+), and no growth (-). (TIF) [file pcbi.1008137.s011.tif]
